# Clinical questions and good practice statements of clinical practice guidelines for management of kidney injury during anticancer drug therapy 2022

**DOI:** 10.1007/s10157-023-02415-0

**Published:** 2023-10-25

**Authors:** Motoko Yanagita, Satoru Muto, Hiroyuki Nishiyama, Yuichi Ando, Sumio Hirata, Kent Doi, Yutaka Fujiwara, Norio Hanafusa, Takahiro Hatta, Junichi Hoshino, Satoko Ichioka, Takamitsu Inoue, Kenji Ishikura, Taigo Kato, Hiroshi Kitamura, Yusuke Kobayashi, Yuichi Koizumi, Chihiro Kondoh, Takeshi Matsubara, Kazuo Matsubara, Koji Matsumoto, Yusuke Okuda, Yuta Okumura, Emiko Sakaida, Yugo Shibagaki, Hideki Shimodaira, Nao Takano, Akiko Uchida, Kimikazu Yakushijin, Takehito Yamamoto, Kazuhiro Yamamoto, Yoshinari Yasuda, Mototsugu Oya, Hirokazu Okada, Masaomi Nangaku, Naoki Kashihara

**Affiliations:** 1 Department of Nephrology, Kyoto University Graduate School of Medicine, Kyoto, Japan; 2 Institute for the Advanced Study of Human Biology (ASHBi), Kyoto University, Kyoto, Japan; 3 Department of Urology, Graduate School of Medicine, Juntendo University, Bunkyo City, Tokyo Japan; 4 Department of Urology, Faculty of Medicine, University of Tsukuba, Tsukuba, Ibaraki Japan; 5 Department of Clinical Oncology and Chemotherapy, Nagoya University Hospital, Nagoya, Japan; 6 Department of Academic Education, I and H Co., Ltd, Ashiya, Japan; 7 Department of Emergency and Critical Care Medicine, The University of Tokyo Hospital, Bunkyo City, Tokyo Japan; 8 Department of Thoracic Oncology, Aichi Cancer Center, Nagoya, Japan; 9 Department of Blood Purification, Tokyo Women’s Medical University, Shinjuku City, Tokyo Japan; 10 Department of Respiratory Medicine, Anjo Kosei Hospital, Anjo, Japan; 11 Department of Nephrology, Tokyo Women’s Medical University, Shinjuku City, Tokyo Japan; 12 Department of Pediatrics, Shiga University of Medical Science, Otsu, Shiga Japan; 13 Department of Renal and Urologic Surgery, International University of Health and Welfare Narita Hospital, Chiba, Japan; 14 Department of Pediatrics, Kitasato University School of Medicine, Minato, Kanagawa Japan; 15 Department of Urology, Osaka University Graduate School of Medicine, Osaka, Japan; 16 Department of Urology, Faculty of Medicine, University of Toyama, Toyama, Japan; 17 Department of Obstetrics and Gynecology, Keio University School of Medicine, Minato, Tokyo Japan; 18 Department of Pharmacy, Seichokai Fuchu Hospital, Izumi, Japan; 19 Departments of Medical Oncology, National Cancer Center Hospital East, Kashiwa, Japan; 20 Department of Pharmacy, Wakayama Medical University Hospital, Wakayama, Japan; 21 Hyogo Cancer Center, Akashi, Hyogo Japan; 22 Department of Gastrointestinal and Medical Oncology, National Hospital Organization Kyushu Cancer Center, Fukuoka, Japan; 23 Department of Hematology, Chiba University Hospital, Chiba, Japan; 24 Division of Nephrology and Hypertension, Department of Internal Medicine, Saint Marianna University School of Medicine, Kawasaki, Kanagawa Japan; 25 Division of Medical Oncology, Faculty of Medicine, Tohoku Medical and Pharmaceutical University, Sendai, Japan; 26 Department of Gastroenterological Surgery (Surgery II), Nagoya University Graduate School of Medicine, Nagoya, Japan; 27 Department of Nursing, Seirei Sakura Citizen Hospital, Chiba, Japan; 28 Department of Medical Oncology and Hematology, Kobe University Hospital, Kobe, Hyogo Japan; 29 Department of Pharmacy, The University of Tokyo Hospital, Bunkyo City, Tokyo Japan; 30 Department of Pharmacy, Kobe University Hospital, Kobe, Japan; 31 Department of Nephrology, Internal Medicine, Nagoya University Graduate School of Medicine, Nagoya, Japan; 32 Department of Urology, Keio University School of Medicine, Minato, Tokyo Japan; 33 Department of Nephrology, Saitama Medical University, Saitama, Japan; 34 Division of Nephrology and Endocrinology, The University of Tokyo, Bunkyo City, Tokyo Japan; 35 Department of Nephrology and Hypertension, Kawasaki Medical School, Kurashiki, Japan

**Keywords:** Glomerular filtration rate, Acute kidney injury, Anticancer drug therapy, Immune checkpoint inhibitors, Cancer survivors

## Guideline information

The main purpose of the “Clinical Practice Guidelines for Management of Kidney Injury During Anticancer Drug Therapy 2022” is to answer as specifically as possible the questions that medical professionals, who are involved in treating various kidney injuries that occur during cancer pharmacotherapy, often encounter in their daily practice and to convey the current standard modes of thinking and specific details of medical treatments to support them in the clinical decision-making. It should be noted that these guidelines are not intended to provide standards of judgment in medical disputes or litigation.

These guidelines cover renal function assessment, management of drug-induced nephrotoxicity, anticancer drug regimen planning, and chronic kidney disease (CKD) treatment in cancer survivors. Additionally, although these guidelines were created from an individual perspective, they do not uniformly recommend a policy that follows the recommendations. The intended users and institutions are medical teams and institutions, including all medical professionals involved in cancer pharmacotherapy.

The Japanese Society of Nephrology (JSN), Japan Society of Clinical Oncology (JSCO), Japanese Society of Medical Oncology (JSMO), and Japanese Society of Nephrology and Pharmacotherapy (JSNP) are responsible for the content of these guidelines, although the physician who is directly involved in the treatment, will assume the responsibility for individual patients.

The JSN provided funding for the preparation of these guidelines. The funds were used for literature search and acquisition, reprint license fees, application fees, and rewards for cooperating lecturers. There was no remuneration for the guideline drafting committee members, systematic review (SR) committee members, questionnaire drafting committee members, or external committee members. No part of the process of preparing these guidelines was funded by any party whose nature could cause conflicts of interest. Conflicts of interest of all committee members involved in the preparation are disclosed in the book [1].

These guidelines comprise “general reviews” that explain background questions common to various medical fields and Clinical questions (CQs) that are clinical foreground questions. First, 16 CQs of the old guidelines were reviewed at a general meeting of the drafting group: those that were widely recognized as effective after publication of the old guidelines and those for which it was concluded that future clinical trials are unlikely to be included in the Good Practice Statement (GPS). Next, the CQs in the old guidelines that were deemed to have relatively low priority to conduct SR again were incorporated into the “general review” with the recommendation grade removed. Furthermore, to minimize conflicts of interest, new CQs were established through consensus building within the committee, such that the intentions of specific members would not be reflected. Ultimately, 16 general reviews, four GPSs including two from the CQs in the old guidelines and the two new GPSs, and 11 CQs including four from CQs in the old guidelines and seven new ones, were adopted.

The databases used for literature search were PubMed®, Cochrane Library, and ICHUSHI-web databases and the search period was set from January 1, 1970, to March 31, 2021. The PICO (Problem/Population, Intervention, Comparison, Outcome) format was used for the literature search. While selecting and excluding literature, we prioritized clinical practice guidelines and SR papers, if they existed; however, if they did not, SR was performed on individual research papers. In such cases, we searched in order of randomized controlled trials (RCTs), non-RCTs, and observational studies. Case reports were excluded. The literature search formula was fixed in April 2021; the primary results of the SR were submitted in July 2021, and the final results of the SR were submitted in September 2021. The literature search formula and SR results for each CQ were published online (https://jsn.or.jp/medic/guideline/gl2022-document.php). All literature searches were performed by the Japan Medical Library Association.

We assessed the certainty of the evidence according to the Minds Manual for Guideline Development 2017 [2] and used the Cochrane Assessment Tool to assess the risk of bias in individual studies. The overall certainty of the evidence was described in the following four grades: (A: strong), (B: moderate), (C: weak), and (D: very weak).

In determining the recommendation grade, we held voting by a consensus development committee that included, in addition to guideline development committee members, external evaluation members such as patient association representatives and physicians, pharmacists, and nurses belonging to related societies other than the drafting organization (Japanese Society for Dialysis Therapy and Japan Academy of Nephrology Nursing), and we described the reason for the resulting judgment and agreement rate of voting. If 75% or more voters agreed, the strength of the recommendation was considered agreed upon, and if less than 75% voters agreed, the results were announced, the recommended proposal was revised, and a second vote was held. If there was no agreement on the recommendation even after repeating this process twice, “not graded” was assigned for the corresponding CQ. As a general rule, we decided to recommend the standard treatment in Japan, although we do not necessarily focus on insurance coverage. The strength of the recommendation was described in one of four classes, namely (1) strongly recommended (to perform), (2) weakly recommended (proposed to perform), (3) weakly recommended (proposed to not perform), and (4) strongly recommended (not to perform).

After preparing the draft, we asked the Japanese Society for Dialysis Therapy to review the manuscript draft, in addition to the four societies that drafted it. Additionally, the draft was published on the websites of these societies to invite public comments. Finally, after revising the draft based on these evaluations, the guidelines were approved by the boards of directors of JSN, JSCO, JSMO, and JSNP. When translating these guidelines into English, 11 CQs and four GPSs were combined into one article, and the remaining 16 reviews were edited into four articles. This article presents an English translation of the 11 CQs and four GPS in the revised edition. We hope that the revised edition will be of assistance to daily clinical practice.

## CQ1: Is the use of estimation formulae recommended for assessing renal function (Glomerular Filtration Rate; GFR) in patients with cancer?

It is recommended to use a GFR estimation formula based on serum creatinine (Cr) levels to evaluate renal function (GFR) before and after administration of cancer pharmacotherapy while considering its limitations. The GFR estimation formula developed by the Japanese Society of Nephrology is useful for Japanese patients. However, the GFR should also be measured in patients with significant deviation in muscle mass from the standard muscle mass and in those with significant weight loss during treatment. In Japan, Inulin clearance is available as a measure of the GFR.

**Recommendation grade**: Strongly recommended (agreement rate: 85.2%, votes: 27 voters, agreement: 23 voters).

## Values and preferences associated with recommendations

While preparing the recommendation for this CQ, we emphasized the convenience of evaluating renal function in daily medical care. To accurately evaluate renal function, the GFR should be measured by inulin clearance; however, it is difficult to measure the GFR quickly and repeatedly during each outpatient visit. Therefore, we evaluated whether the estimated GFR (eGFR) value can be used as a substitute for the actual GFR measurements.

The outcome of this CQ is the degree of approximation, that is, the accuracy between the measured and reference eGFR values. We used P30, defined as the “percentage of estimates within 30% of the measured GFR,” as the method of evaluation. P30 can evaluate the GFR estimation formula has been reported in systematic reviews of patients with diabetes, those who have undergone renal transplantation, and those with obesity and is also recommended by the guidelines of the National Kidney Foundation (Kidney Disease Outcomes Quality Initiative, KDOQI) and KDIGO (Kidney Disease Improving Global Outcomes) guidelines [3–7]. As an interpretation of P30, the KDOQI considers a P30 value of ≥ 90% to be appropriate and a value of ≥ 75% to be sufficient for clinical decision-making [3]. The KDIGO guidelines also state that the appropriate value of P30 is ≥ 90%; however, there are only few estimation formulae that satisfy this criteria [4, 8].

## Summary of evidence for CQ

[Certainty of evidence: C (weak)]

## Evaluation items to determine the strength of the recommendation

1. Factors that influence the determination of the strength of the recommendation

**There is strong overall evidence for the overall outcome[Assessment: No]**

The publication by Funakoshi et al. [9] was the only study to evaluate the estimation formula of the Japanese Society of Nephrology with P30 as the outcome. Therefore, study using the Chronic Kidney Disease Epidemiology Collaboration (CKD-EPI) formula was the subject of the meta-analysis, although this estimation formula is used overseas and does not apply to Japanese patients. Additionally, P30 adopted for evaluating the outcome did not reach 90% as recommended by the KDOQI and KDIGO. Funakoshi et al. compared measured GFR values with eGFR values obtained using the estimation formula of the JSN in 50 patients with cancer and found that P30 was 92%.

**The benefit-harm balance is certain [Assessment: Yes]**

Measuring the GFR at every time-point in the evaluation of renal function increases the burden on patients and healthcare providers. If the actual measurements of GFR are mandatory to evaluate renal function, the number of evaluations is likely to decrease due to the difficulty of measurement, and this will cause significant harm to medical care.

2. 2. Factors to consider for the strength of the recommendation

For patients, GFR measurements using inulin clearance (actual measured GFR values) are time-consuming and physically burdensome; thus, it is preferable to use eGFR values that can be calculated from a single blood sample. Additionally, the use of eGFR values is preferred because measuring inulin clearance is more expensive than measuring the serum Cr value for the out-of-pocket portion of medical expenses.

## Commentary on recommendation

## Background and purpose

As the number of patients with CKD increases, there is concomitant increase in the number of CKD patients treated with cancer pharmacotherapy. Furthermore, cancer pharmacotherapy may also cause acute or chronic kidney damage. Additionally, with the advancement of cancer pharmacotherapy, it has become possible to prolong the lifespan of patients with advanced cancer; however, novel renal adverse events are emerging with advent of molecular targeted therapeutics and immune checkpoint inhibitors (ICIs). Drugs cleared by renal excretion require evaluation of renal function during dose setting. Therefore, accurate evaluation of renal function is important for risk assessment before treatment, drug dose setting, and early diagnosis and treatment of kidney damage associated with pharmacotherapy.

Renal functions (including hemofiltration in the glomerulus, substance transport in the renal tubules, and metabolic and endocrine functions) are diverse. The GFR is used for the diagnosis and classification of acute kidney injury (AKI) and CKD to quantitatively evaluate renal function. Drug dose adjustment is often based on the GFR or creatinine clearance (CCr) that reflects the GFR. To accurately evaluate the GFR, inulin clearance and/or nuclear medicine assessments using radioisotopes are necessary. However, these methods are complicated and expensive, making them difficult to perform as routine tests. Alternatively, GFR estimation formulae based on serum creatinine and cystatin C have been developed and are widely used in the diagnosis and classification of CKD. However, GFR estimation formulae that are generally used (such as CKD-EPI and GFR estimation formulae developed by the JSN) are not designed for patients with cancer; thus, their validity for patients receiving cancer pharmacotherapy remain uncertain.

## Target and method

Therefore, in response to the CQ "Is the use of estimation formulae recommended for assessing renal function (GFR) in patients with cancer?" we conducted a SR of the accuracy assessment of eGFR values, using measured GFR values as controls. The outcome was the accuracy of the eGFR value; and P30, which is defined as “the ratio of cases where the eGFR value is within ± 30% of the measured GFR value,” was used as the method of evaluation. P30 is used in systematic reviews evaluating GFR estimation formulae in patients with diabetes, those who have undergone kidney transplantation, and those with cirrhosis, and it is recommended as a method of evaluation for GFR estimation in the KDOQI and KDIGO guidelines.

By sending a request to the Japan Medical Library Association, we had papers that evaluated measured GFR and eGFR using P30 extracted from the databases of PubMed, Cochrane, and the Japan Medical Abstracts Society. The two reports by Funakoshi et al. were the only publications that studied Japanese patients with cancer and GFR estimation formulae developed by the JSN [9, 10], while most of other papers evaluated CKD-EPI, Modification of Diet in Renal Disease (MDRD), and Cockcroft-Gault developed in the United States. Therefore, a meta-analysis was conducted on nine papers evaluating the accuracy of the CKD-EPI formula, which is currently the most widely used worldwide [10–18]. The collective estimate was 81% (95% confidence interval (CI): 71–91%). The KDOQI considered a P30 value of ≥ 75% to be sufficient for clinical decision, while the recommended target is ≥ 90%. The result of the meta-analysis of the nine papers in this study was 81%; thus, it was considered appropriate. The I^2^ value, which shows heterogeneity, was as high as 97.9%; notably, the characteristics of the patients (race and type of cancer), and method of GFR measurement varied among reports. Based on these factors, the strength of the evidence was deemed weak.

## Comparison of eGFR and measured GFR

The studies by Funakoshi et al. on the accuracy of the eGFR in Japanese patients with cancer were the only ones to compare the eGFR to actual measured GFR values [9, 10], while others made comparisons with 24-h CCr. Inoue et al. [19] studied the ratio of eGFR overestimated by ≥ 30% than the measured GFR; however, we did not include their study in this meta-analysis because their control was CCr rather than the measured GFR value, and they did not include cases where the eGFR was lower than the measured GFR value. Funakoshi et al. compared the GFR values measured by inulin clearance with the CKD-EPI formula, the estimation formula of the Japanese Society of Nephrology, Cockcroft-Gault formula, and CCr obtained using 24-h urine specimens collected from 50 patients with cancer; the accuracy assessed by P30 was 92% for the formula developed by the JSN and the CKD-EPI formula. Funakoshi et al*.* published two papers, both of which included the same patient population and one examined data before and after cisplatin administration; thus, we partly used the data before cisplatin from this paper for the meta-analysis.

## GFR measurement method and evaluation of eGFR

Inulin clearance measurement is covered by insurance, and the required reagents are commercially available in Japan. Measurement requires intravenous (IV) administration of an inulin reagent and collection of urine over time; thus, it is difficult to perform this as a routine examination in daily clinical practice. However, this approach requires no special equipment other than an insulin-dissolving device, and it can be performed without a special facility.

In future research on renal function evaluation of Japanese patients with cancer, we would like to propose GFR measurement by inulin clearance or nuclear medicine examination as a control; moreover, we would like to propose utilizing P30 for evaluating GFR estimation.

## Conclusions and challenges

Based on the above, it is recommended to use the eGFR value based on the serum Cr value, which can be easily and quickly measured, for evaluation of renal function before pharmacotherapy and during the course of treatment in routine clinical practice. When using it, one should recognize the limit of the eGFR value, pay attention to its interpretation among patients whose body sizes deviate significantly from the standard physique, such as due to emaciation, and actual GFR measurement or other estimation equations should be considered concurrently, if necessary.

## CQ2: Is it recommended to use novel AKI biomarkers for early diagnosis of AKI due to anticancer drugs such as cisplatin?

AKI is often diagnosed around the third day after cisplatin administration. Studies suggest that the onset of AKI can be predicted more than a day in advance by measuring novel AKI biomarkers such as neutrophil gelatinase-associated lipocalin (NGAL), in urine. Although early diagnosis of AKI can raise awareness, there is no report suggesting that it can improve renal or vital prognosis, and the benefit of novel AKI biomarker measurement is limited. There have not been many reports that attempted to analyze early diagnosis of AKI in treatment with molecular targeted therapeutics.

**Recommendation grade**: Weakly recommended (proposed) (agreement rate: 100%, votes: 27 voters, agreement: 27 voters).

## Values and preferences associated with recommendations

Several studies [20–24] have shown that measurement of novel AKI biomarkers can predict the onset of cisplatin-induced AKI more than a day before the diagnosis by serum Cr levels, suggesting evidence for early diagnosis. In Japan, among these biomarkers, measurement of urinary L-type fatty acid-binding protein (L-FABP) and NGAL is covered by insurance for patients with suspected AKI, while measurement of kidney injury molecule-1 (KIM-1), and NephroCheck® (urinary tissue inhibitor of metalloproteinase 2 multiplied by insulin-like growth factor-binding protein 7 concentration) are not. Measurement methods and cut-off values vary among reports, and operation and interpretation of measurements depend on individual institution.

Whether early diagnosis of cisplatin-induced AKI by real-time measurement of these biomarkers can improve renal and vital prognosis has not been investigated, and it is not clear whether there are significant clinical benefits from biomarker measurement. However, it can be interpreted that early diagnosis of AKI can raise awareness.

## Summary of evidence for CQ

[Certainty of evidence: C (weak)].

## Evaluation items to determine the strength of the recommendation

1. Factors that influence the determination of the strength of the recommendation

**There is strong overall evidence for the overall outcome [Assessment: Yes]**

There is evidence that multiple markers contribute to the early diagnosis of AKI, implying strong overall evidence.

**The benefit-harm balance is certain [Assessment: No]**

The benefits of early diagnosis are not significant.

2. Factors to consider for the strength of the recommendation

Although assessments using novel AKI biomarkers before and after cisplatin treatment are useful for the early diagnosis of AKI, it is very difficult to predict renal function adequately before cisplatin administration.

## Commentary on recommendation

## Background and purpose

Cisplatin is a widely used anticancer drug, although its side effects such as AKI, hypomagnesemia, and hypokalemia should be considered [25, 26]. Urinary L-FABP and NGAL are novel AKI biomarkers for early diagnosis of AKI after cardiovascular surgery and in the intensive care units [27, 28]. Whether novel AKI biomarkers are useful for early diagnosis of drug-induced nephrotoxicity (DIN) or AKI due to anticancer drugs such as cisplatin, ICIs, and other molecular targeted therapeutics is an important clinical challenge.

Following cisplatin treatments, AKI occurs in approximately 30% cases, with serum Cr levels starting to rise from 3 days after cisplatin administration and peaking at 6–10 days [25, 26, 29, 30]. Serum Cr level could peak earlier in milder renal disfunction. When cisplatin is administered, patients often undergo large amounts of hydration to minimize its nephrotoxicity, and are less likely to cause oliguria [31], or require acute blood purification [26, 31–33]. An increase of > 50% in serum Cr levels 3 or 4 days after cisplatin administration is often used as an actual diagnostic criterion for AKI [20–22, 34, 35].

## Target

The SR of this CQ considered five bio-molecules [36], including urinary L-FABP and NGAL, which have been listed in Japan’s national health insurance since 2000, as well as urinary KIM-1, interleukin-18, and NephroCheck, which have been introduced in the 2016 version of this guideline and widely applied in clinical research and practice worldwide. Urinary albumin and N-acetylglucosaminidase were not included.

## AKI prediction after cisplatin administration

Publications up to March 2021 were screened in the databases of PubMed, Cochrane, and the Japan Medical Abstracts Society using the keywords AKI, biomarkers (the above-mentioned five bio-molecules), and cisplatin. Furthermore, we investigated whether the onset of AKI after cisplatin administration could be predicted at least 1 day before the serum Cr level increase by measuring a novel AKI biomarker. We found five reports suggesting that diagnostic accuracy can be evaluated qualitatively based on sensitivity and specificity. Early diagnosis was deemed possible by urinary NGAL in three reports [21–23], by KIM-1 in three reports [21, 22, 24], and by NephroCheck in one report [20], although there are duplicate references. (Forest plots are posted in the supplementary material "CQ2_SR template" that can be downloaded from the web (https://jsn.or.jp/medic/guideline/gl2022-document.php).

Some reports suggested that early diagnosis is possible, although qualitative evaluation is not possible [29, 31, 37], while others suggested that early diagnosis is not possible [30, 32, 38, 39]. An abstract from a conference report suggested that early diagnosis can be achieved using urinary L-FABP [37].

## Changes in biomarkers over time

Considering changes in biomarkers over time, the urinary NGAL levels or NGAL/Cr increased significantly between 12 h and 3 days after cisplatin administration in patients with AKI [29, 33]. The urinary KIM-1 levels also increased 1 to 3 days after cisplatin administration in patients with AKI [21, 24]. The NephroCheck levels were elevated in urine collected within 12 h after cisplatin administration from patients with AKI [20]. When seven analyses involving a total of 421 cases were combined to examine the usefulness of biomarkers, the positive clinical usefulness index (CUI) value was 0.782 (0.64–0.81; indicating good utility), while the negative CUI value was 0.915 (≥ 0.81; indicating excellent utility) [40].

However, the timing and method of measurement, as well as the cut-off values of biomarkers are inconsistent. Additionally, the increase in biomarkers was measured using the absolute value [20, 21, 24], values corrected by urinary Cr [23, 31], and rate of change from the previous value [22, 29]. Urinary NGAL levels tend to be high in patients with urinary tract infection; thus, it is useful to check the leukocyte count and presence or absence of bacteria by urinary sedimentation to differentiate the above-mentioned patients from those with AKI [41].

Further, because cisplatin administration is accompanied by large amount of hydration to minimize its nephrotoxicity, comparisons of urinary biomarkers described above between pre- and post-treatment should use corrected urinary Cr levels rather than the absolute value. If AKI is suspected based on the urinary NGAL level, the calculation should be performed once at the time of diagnosis, and thereafter up to three times per series of treatments for AKI. If calculations are further performed due to medical necessity, the detailed reason for this should be stated in the Description column of the Medical Fee Statement.

## Drugs other than cisplatin

There were not enough reports on drugs other than cisplatin. The prevalence of AKI caused by carboplatin is low. Among the studies that calculated the incidence of AKI due to the use of platinum-based anticancer drugs, one report indicated that AKI occurred in 31% (22/71) of cisplatin cases and 20% (1/5) of carboplatin cases [21], while another reported that AKI occurred in 17% (4/24) of cisplatin cases and 0% (0/8 cases) of carboplatin cases [20]. Since DIN due to ICIs and other molecular targeted therapeutics develops several weeks or months after repeated administration, it was assumed that a research design of repeated measurement of novel AKI biomarkers and to predict DIN would be difficult to establish. Indeed, a literature search failed to reveal any reports that examined the early diagnosis of AKI due to these anticancer drugs.

## Conclusions and challenges

To prove early diagnosis of AKI using novel biomarkers to be clinically useful, it is necessary to demonstrate differences in renal and vital prognoses between populations that have or have not undergone measurement of novel biomarkers. However, such research has not yet been conducted sufficiently, even considering studies on AKI in general. Therefore, in this CQ, AKI prediction before diagnosis by serum Cr levels was considered an important outcome.

Although no drugs have been established as therapeutic agents for renal AKI, including cisplatin-induced AKI [28], if AKI is diagnosed at an early stage, it would be possible to take measures, such as careful and frequent observation and hemodynamic monitoring to maintain body fluid volume and renal blood flow, while avoiding the administration of nephrotoxic drugs [35].

Understanding whether the measured values of novel AKI biomarkers provide information for deciding whether to reduce the dose of anticancer drugs and repeat administration or to switch to another drug when mild AKI occurs with anticancer drugs is a long-term challenge.

## CQ3: If hydronephrosis is present before cancer treatments, is it recommended to perform a ureteral stent placement or nephrostomy?

Ureteral stent placement or nephrostomy should be performed for improving renal function in case of post-renal kidney dysfunction due to malignant ureteral obstruction (MUO), considering that it will be accompanied by deterioration of quality of life (QOL). However, in cases of mild renal function impairment, there is no clear evidence of prolonged survival resulting from ureteral stent placement or nephrostomy (intended for improving renal function during cancer drug therapy); thus, indications should be determined by considering the expected survival period and possibility of deterioration in QOL for individual patients with each type of malignancy.

**Recommendation grade**: Strongly recommended (agreement rate: 100%, votes: 28 voters, agreement: 28 voters).

Values and preferences associated with recommendations. (Assume a set of values for each outcome considered)

Considering the prolongation of overall survival (OS) and progression-free survival (PFS), there are only two reports on patients with cervical cancer with low evidence levels in comparison with a control group [42, 43], and it is unclear whether these results can be applied to other types of cancer.

These studies do not differentiate between bilateral or unilateral MUO. In cases of bilateral MUO, rescue from post-renal acute renal failure by performing ureteral stent placement or nephrostomy may be expected to have a “large effect” on OS and should be considered in determining the certainty of the evidence.

There have been no studies of comparison with a non-intervention group on whether performing a ureteral stent placement or nephrostomy would improve the eGFR of < 60 mL/min/1.73 m^2^ (cisplatin-unfit) to a value of ≧60 mL/min/1.73 m^2^ (cisplatin-fit) in patients with unilateral MUO, where anticancer drugs were nephrotoxic and dose reduction are required based on renal function. However, there have been retrospective studies observing renal function over time after the intervention. It is unclear whether this leads to the prolongation of OS or PFS in all types of malignancy because there are no publications available to evaluate the evidence.

Regarding QOL improvement, there has been only one report with a weak certainty of evidence in patients with cervical cancer, mentioning that placing a ureteric stent would be more harmful than non-intervention. Anatomically being adjacent, direct infiltration of bladder from cervical cancer may strongly influence the results; although it is unclear whether this result can be applied to other types of malignancies. However, it is important to determine whether intervention for MUO rescue should be performed considering the balance between prognosis and QOL for any type of malignancy.

Although this CQ3 focuses on a problem in daily medical care, the evidence in this field are still inadequate. Although we provided a “Strongly recommended” recommendation considering the current clinical situation, it is strongly recommended that the approach is chosen individually for each patient by considering the following situations; whether MUO is unilateral or bilateral, whether long-term prognosis is expected, whether the drugs planning to be used can cause renal dysfunction, whether the eGFR is ≧60 mL/min/1.73 m^2^ or < 60 mL/min/1.73 m^2^, whether continued cancer pharmacotherapy should be considered despite the expected eGFR decline in future, and whether the QOL would be affected.

## Summary of evidence for CQ

[Certainty of evidence: C (weak)].

## Evaluation items to determine the strength of the recommendation

1. Factors that influence the determination of the strength of the recommendation

**There is strong overall evidence for the overall outcome [Assessment: No]**

The certainty of evidence is C (weak).

**The benefit-harm balance is certain [Assessment: No]**

There are reports that provide weak evidence for benefits regarding OS and PFS; there are also reports that provide weak evidence for harm regarding QOL improvement. Regarding bilateral MUO, patients can expect a “large effect of intervention (ureteral stents placement or nephrostomy)” for post-renal acute renal failure on OS, which would significantly influence the certainty of evidence.

2. Factors to consider for the strength of the recommendation

In the case of unilateral MUO with mild kidney dysfunction, dose reduction based on renal function is necessary for nephrotoxic anticancer drugs. Conversely, there is no evidence of improved survival by performing a ureteral stent placement or nephrostomy in patients with eGFR of < 60 mL/min/1.73 m^2^; and it is impossible to assert the “large effect of intervention” in these patients. Patients’ (families’) intentions for this treatment should differ significantly with respect to QOL between ureteral stent placement and nephrostomy. Regarding the ureteral stent placement, the patient burden in terms of cost and QOL is relatively low, and the treatment is considered more acceptable. There is one report of cervical cancer providing weak evidence regarding the harmful effects of intervention being compared with a non-intervention group. Regarding nephrostomy, the need for hospitalization during treatment, patient’s QOL burden associated with the need for post-treatment nursing care, and usage of social resources such as home-visit nursing care make it unlikely for patients to be receptive to the intervention.

## Commentary on recommendation

## Objective outcome of ureteral stent placement or nephrostomy

There may be situations such that the administration of the intended pharmacotherapy would be questioned for patients whose renal function is already compromised or predicted to decline by unilateral or bilateral hydronephrosis due to lymph node metastasis or peritoneal dissemination, diagnosed in the imaging before cancer pharmacotherapy The rescue of MUO for post-renal nephropathy before cancer pharmacotherapy in these patients, provided by performing unilateral or bilateral ureteral stent placement or nephrostomy, is a matter requiring important clinical judgment. The intended outcomes of these renal function-preserving interventions are as follows; enablement of continuous administration of nephrotoxic anticancer drugs such as cisplatin, elimination of dose reduction in anticancer drugs in the short-term outcome, improvement in the response rate to cancer pharmacotherapy, prolongation of PFS, OS, and improvement in QOL in the long-term outcome.

## Results of the systematic review

We found no reports of prospective RCTs comparing the above-mentioned outcomes between the non-intervention and intervention (ureteral stent placement or nephrostomy) groups to rescue MUO in any area of malignancy. In patients with cervical cancer, there have been two retrospective cohort studies with weak certainty of evidence on OS and PFS, and only one on QOL. Nevertheless, careful interpretation is needed to determine whether these results can be applied to other types of malignancy because the malignancy sites and characteristics differ. There are no comparative studies for cisplatin-unfit patients to determine whether the interventions (ureteral stent placement or nephrostomy) improves eGFR to cisplatin-fit levels over time and prolong survival when compared with non-intervention patients; however, there have been two observational studies on the changes in renal function over time [42, 43].

## Evidence of prolonged survival

Considering OS and PFS, there is a prospective cohort study that compared the OS of 230 cervical MUO; 49 patients did not require the rescue intervention (serum Cr level ≦150 μmol/L [1.7 mg/dL] and in the normal range), 93 underwent the rescue intervention, and 56 needed but did not undergo the rescue intervention [44]. The results showed that OS was longer in the following order: arm not requiring the rescue intervention, arm receiving the rescue intervention, and arm requiring but not receiving the rescue intervention; the results associated with the log-rank method showed significant differences between the three groups [44]. It is noteworthy that the prognosis of the arm receiving the rescue intervention improved more than that of the arm requiring but not receiving the rescue intervention, despite significantly obvious pre-treatment hydronephrosis, a higher proportion of bilateral hydronephrosis, and a higher serum Cr level.

Another cohort study analyzed four prospective randomized trials conducted by the Gynecologic Oncology Group in the United States to investigate retrospectively the differences in OS and PFS with or without hydronephrosis rescue [45]. In these four studies, 539 cervical cancer patients with a serum Cr level of ≦2.0 mg/dL were divided into the following three groups: without hydronephrosis (*n* = 301), with rescued hydronephrosis (*n* = 209), and with unrescued hydronephrosis (*n* = 29) [45]. The results showed that OS and PFS were longer in the following order: the group without hydronephrosis, the group with rescued hydronephrosis, and the group with unrescued hydronephrosis, and analysis by the log-rank method showed significant differences among the three groups. It is noteworthy that there was no significant difference in patient demographics among the three groups, and all patients received protocol-based chemotherapy and radiation therapy [45].

However, the above two papers are retrospective cohort studies, and their conclusions show significant differences in three-group comparisons including those without the rescue intervention and those without hydronephrosis. Therefore, the certainty of evidence regarding OS and PFS is category C, and these interventions are weakly recommended.

## Evidence of QOL improvement

Following the SR on QOL, we identified a retrospective, case–control study comparing the incidence of urinary tract adverse events (AEs) in 1808 cervical cancer patients without metastasis and 5424 controls from the Surveillance, Epidemiology, and End Results (SEER)-Medicare database in the United States, wherein 202 patients had tumors and had undergone ureteral stent placement, 1606 had tumors but had not undergone ureteral stenting, 79 had no tumors and had undergone ureteral stenting, and 5345 had no tumors and had not undergone ureteral stenting [46]. Compared with the group with tumors and no ureteral stent placement, the group with tumors and ureteral stenting showed an increase in prevalence of lower urinary symptoms by 2.79-fold, gross hematuria by 2.76-fold, urinary incontinence by 2.58-fold, urinary retention by 11.21-fold, renal colic by 9.53-fold, urinary calculus by 28.76-fold, and urinary tract infections, including pyelonephritis, by 3.35-fold. There was a significant overall increase in urinary tract AEs. Although the study did not consider the occurrence of hydronephrosis, the incidence of urinary tract AEs in the group with tumors and ureteral stent placement was relatively lower (0.51 to 4.26-fold, respectively for each item) compared to those in the group without tumors and with ureteral stent placement [46]. Additionally, there may be differences in QOL between ureteral stent placement and nephrostomy, although there is no clear evidence currently. Regarding nephrostomy, the burden on the patients due to the need for hospitalization and nursing care, and burden of social resources such as home-visit nursing may be relatively large. Thus, there is a grade C certainty of the evidence for the QOL outcome of these interventions, that is, with respect to QOL, it is weakly recommended not to undergo these interventions.

## Evidence of renal function improvement

Two single-arm observational studies can be used as references to understand whether the interventions (ureteral stent placement or nephrostomy) improve the eGFR from cisplatin-unfit to cisplatin-fit over time. In an observational study of 12 patients with testicular tumor with unilateral hydronephrosis (between 2002 and 2010), unilateral ureteral stent placement (bilateral for one patient) was performed prior to chemotherapy. The mean eGFR improved from 68.3 to 82.5 mL/min/1.73 m^2^; in three patients, the renal function of eGFR of < 60 mL/min/1.73 m^2^ before the intervention improved to an eGFR of ≧60 mL/min/1.73 m^2^ after the intervention, thus allowing patients to receive chemotherapy without dose reduction [41]. Additionally, 87 patients who had undergone bilateral ureteral stent placement (14 with non-malignant tumors) for bilateral MUO caused by various malignancies were observed retrospectively; the ratio of patients with CKD stage 3 or higher (eGFR < 60 mL/min/1.73 m^2^) decreased from 80.5% before stent placement to 54.1% 6 months after stent placement [43].

## The reason for a strong recommendation despite the weak certainty of each piece of evidence

The rationale and conclusions for the strong recommendation despite the weak certainty of evidence are that the comparative studies of OS and PFS introduced above did not distinguish between bilateral or unilateral MUO. For bilateral MUO, prolongation of OS by relieving post-renal acute renal failure through ureteral stent placement or nephrostomy is strongly anticipated to have a “large effect of intervention;” thus, considering the certainty of evidence, ureteral stent placement and nephrostomy can be strongly recommended interventions.

There is evidence of weak certainty in observational studies of intervention in the situation of “the doses of anticancer drugs that are nephrotoxic need to be reduced because the eGFR is < 60 mL/min/1.73 m^2^ before pharmacotherapy due to unilateral MUO or it is predicted to decline to an eGFR < 60 mL/min/1.73 m^2^ in the future.” However, there is no certain evidence that for all types of malignancy the improvement of renal function makes cancer pharmacotherapy possible or eliminates the necessity to dose reduction, leading to the improvement of the response rate and prolongation of OS and PFS. Therefore, this intervention can be weakly recommended.

For the general recommendations, there is weak evidence that ureteral stent placement or nephrostomy is beneficial for OS and PFS compared with non-intervention groups, and there is weak evidence of the harmful effects of interventions on QOL improvement in patients with cervical cancer. There is no evidence for OS and PFS in comparison with non-intervention populations in any other malignancy. Despite the contradictory and weak evidence, there is no evidence to deny the "large effect of intervention” of ureteral stent placement and nephrostomy, particularly for bilateral MUO. Therefore, considering the current clinical situation, we “Strongly recommend” this intervention.

## Future challenges

Although the field of this CQ3 focuses on a problem in daily medical care, it has become clear that the evidence is still insufficient. To clarify whether immediate rescue of unilateral MUO makes cancer pharmacotherapy feasible, eliminates the dose reduction of anticancer drugs, improves the response rate of cancer pharmacotherapy, and prolongs OS and PFS, it is necessary to perform comparative studies with delayed rescue of MUO. Additionally, the following several studies could be anticipated; the comparison of the relationship between MUO rescue, overall survival, and deterioration of QOL in each type of malignancy, a comparison of the deterioration of QOL between ureteral stent placement and nephrostomy, and the analysis evaluating whether only unilateral rescue is sufficient in patients with post-renal acute renal failure due to bilateral MUO.

## CQ4: Is the use of immune checkpoint inhibitors (ICIs) recommended in hemodialysis patients?

The use of ICIs in hemodialysis patients is recommended because of the availability of safety information to a certain level and possibility of a higher response rate of renal cell carcinoma to ICIs than to molecular targeted therapy. This recommendation is based on the accumulation of case reports on the use of ICIs in hemodialysis patients.

**Recommendation grade**: Weakly recommended (proposed) (agreement rate: 92.6%, votes: 27 voters, agreement: 25 voters).

Values and preferences associated with recommendations (Assuming a set of values for each outcome considered)

It is still recent for ICIs to be commonly used in cancer chemotherapy, and there are few studies that can provide evidence on the use of ICIs in hemodialysis patients. Owing to insufficient observational studies, it is necessary to refer to the accumulated information from case reports, and thus, the outcomes that can be evaluated are limited. Additionally, it is necessary to specify the type of cancer to compare outcomes; thus, this CQ focuses on renal cell carcinoma, which has a large number of reported cases.

OS and PFS with ICIs are the most important outcomes for patients with cancer. However, we did not evaluate them because in many cases, it was not possible to accurately collect this information from case reports.

The response rate is a beneficial outcome available from case reports; although, generally less important than OS, the fact that the response rate for ICIs was higher than that to molecular targeted therapy, it was considered beneficial.

While the incidence of an immune-related adverse event (irAE) is an unfavorable outcome available from case reports, it was difficult to evaluate because there is no direct comparator. It was considered beneficial that the incidence of ≥ grade 3 AEs from ICI tended to be lower compared with those from molecular targeted therapies.

There were no significant differences in the response rate to ICIs and incidence rate of irAEs in hemodialysis patients compared with the general population without renal replacement therapy; therefore, the use of ICIs was evaluated as not harmful in hemodialysis patients.

## Summary of evidence for CQ

[Certainty of evidence: D (very weak)].

## Evaluation items to determine the strength of the recommendation

1. Factors that influence the determination of the strength of the recommendation

**There is strong overall evidence for the overall outcome [Assessment: No]**

The certainty of the evidence is D (accumulation of case reports). As it was difficult to evaluate OS, the certainty of evidence was judged solely on the response rate to ICIs and incidence rates of AEs.

**The benefit-harm balance is certain [Assessment: No]**

The response rate to ICIs, which is the outcome of the benefit, tends to be higher than that of other treatments for renal cell carcinoma in hemodialysis patients. The incidence of an irAE has been reported as a harmful outcome; despite the absence of comparison, the incidence rates of irAEs in hemodialysis patients were almost the same as those in non-hemodialysis patients. Although we concluded that the benefits possibly outweigh the harmful effects, the outcomes that can be evaluated are limited here.

2. 2. Factors to consider for the strength of the recommendation

ICIs have risk of irAEs, which do not occur with the use of cytotoxic anticancer drugs and molecular targeted therapies. IrAE is an AE that requires extreme caution and, in severe cases, can be fatal. How patients perceive this risk is greatly influenced by their sense of values. The extent to which patients consider the cost-effectiveness of ICIs with relation to the survival benefits of these drugs depends on their sense of values.

## Commentary on recommendation

## Background and purpose

Hemodialysis patients in Japan have a higher incidence of certain cancers than the general population [47]. In addition to general risk factors such as age, cancer is often detected in the early stages of hemodialysis induction, suggesting that risk factors attributable to CKD may be related to the incidence of cancer. In dialysis patients, the standardized incidence ratio (SIR) of urological cancers such as renal cell carcinoma and urothelial cancer is high [48]. In addition, considering the approval status of ICIs for various indications in Japan, the use of these molecules in patients receiving hemodialysis, becomes an important issue. This is especially significant in Japan where both the dialysis population and number of patients with cancer are increasing due to aging of the population.

## Target

For this CQ, we conducted a SR to compare the usefulness of ICIs with other treatment modalities administered to hemodialysis patients, assuming that this CQ will support such patients in considering their therapeutic options. Although no observational studies were found, 24 case reports, and three reviews based on case reports were found. This CQ study group considered the recommendation based on the information collected through these case reports.

## Survival period

Prolonged OS is the most desired outcome for ICI therapy. Prolongation of PFS is also important because that would strongly lead to its recommendation. Our SR found only case reports and no observational studies; there was insufficient literature that included OS and PFS. Further studies are needed to evaluate these outcomes in this CQ.

## Response rate

As the benefit outcome, we focused on the response rate available from multiple case reports. According to the calculations based on the 24 case reports and 67 patients detected by SR (26 patients with renal cell carcinoma, 9 with melanoma, 8 with lung cancer, 7 with genitourinary cancer, 4 with urothelial carcinoma, and 13 with other cancers), the response rate to ICIs was 46.2% for patients with renal cell carcinoma, which was the most frequently reported type of cancer in hemodialysis patients. For comparison, information accrued through case reports on patients undergoing hemodialysis treated with various molecular targeted therapies, used as first-line treatment of renal cell carcinoma, indicated the response rates to be 33.3% for sunitinib, 4.4% for mammalian target of rapamycin (mTOR) inhibitors, and 15.4% for axitinib [49]. Despite weak certainty because the comparison was based on case report accumulation, the response rate to ICI in patients undergoing hemodialysis was higher than that in those undergoing molecular targeted therapy. Additionally, the response rate for nivolumab monotherapy in the general population with renal cell carcinoma was 25.1% [50] and 41.3% for concomitant nivolumab plus ipilimumab therapy [51], suggesting that hemodialysis patients do not have low response rates to ICI.

## irAEs

The incidence of irAEs is a notable harmful outcome for ICI therapy. According to the calculations based on the 24 case reports and 67 patients detected by SR, 70.1% of cases showed some type of irAE, and 17.9% had grade 3 or higher irAEs. IrAEs are AEs specifically associated with ICIs and cannot be compared to those associated with non-ICI therapy. As a reference, the incidence of AEs in patients receiving hemodialysis treated with molecular targeted therapies and that of irAEs in non-hemodialysis patients treated with ICI are shown. Although limited information is available, the incidence rates of grade 3 or higher AEs in these patients treated with molecular targeted therapies were 26.8% for sunitinib, 23.5% for everolimus, 23.5% for temsirolimus, and 15.4% for axitinib [49]. The incidence rate of irAEs in non-hemodialysis patients was such that in the study of nivolumab monotherapy for patients with renal cell carcinoma mentioned previously, 79% of patients had treatment-related AEs, while 19% had grade 3 or higher serious AEs [50]. Similarly, in a study of concomitant nivolumab plus ipilimumab therapy, treatment-related AEs occurred in 94% of patients, and serious AEs of grade 3 or higher were found in 47% [51]. Although these incidence rates are not directly comparable and vary by type of cancer and drug, hemodialysis patients may not be a population of patients with extremely high incidence of treatment-related AEs, including irAEs.

In this CQ, we could not investigate outcomes related to QOL improvement. Improvement of QOL greatly affects the therapeutic options for patients, and further research is needed.

## Conclusions and challenges

As an overall recommendation, although there are no studies that provide solid evidence, we could evaluate some outcomes for ICI with weak certainty, based on the accrual of case reports. The ICI response rates for hemodialysis patients may be higher than those for standard non-ICI therapies given to patients with renal cell carcinoma. Although there was no comparator, the incidence rate of irAEs in hemodialysis patients was made clear, emphasizing the safety of ICIs. Thus, this CQ study group concluded that ICI therapies can be recommended for hemodialysis patients as the harmful effects of ICI do not outweigh their benefits.

Our review of this CQ used only information from case reports as there was insufficient literature to evaluate the evidence. To ascertain a level of certainty, it is necessary to conduct observational studies with at least the patient demographics being consistent. Additionally, it is necessary to conduct studies that compare outcomes such as OS and PFS between the patients treated with ICI and non-ICI drugs. Furthermore, by analyzing irAEs, which commonly develop in patients undergoing dialysis, it would be possible to identify clinical and laboratory findings monitored for better safety and to build a close cooperation system with medical departments associated with the irAEs.

## CQ5: Is the use of ICIs recommended in patients who have undergone renal transplantation?

The use of ICIs is particularly recommended for squamous cell carcinomas after renal transplantation because ICIs can prolong OS and have a significantly higher response rate than other treatments. Conversely, ICIs can also significantly increase the incidence of kidney rejection in patients after renal transplantation, although a continued use of combination of multiple immunosuppressive drugs, including mTOR inhibitors, may suppress rejection.

**Recommendation grade**: Weakly recommended (proposed) (agreement rate: 100%, votes: 27 voters, agreement: 27 voters).

Values and preferences associated with recommendations (Assuming a set of values for each outcome considered)

ICIs have only recently come into common use in cancer chemotherapy, and there are few published research articles that provide evidence on ICI therapy in patients who have undergone renal transplantation. Since there are limited observational studies on this subject, we believe that information from case reports should be incorporated in the assessment. Additionally, it is necessary to specify the type of cancer to compare outcomes; thus, this CQ focused on melanoma and non-melanoma cutaneous squamous cell carcinoma, which has large numbers of reported cases in patients who have undergone renal transplantation.

Prolongation of OS and PFS with ICIs was evaluated as an important beneficial outcome despite being based on limited observational studies.

Although the response rate is generally less important than OS, it was a limited benefit outcome ascertained from information provided by case reports.

The loss of a transplanted kidney and organ rejection leading to graft loss through ICI therapy are important detrimental outcomes for the transplant recipient, and we emphasized on evaluating them. When the specific ways to avoid such harmful outcomes were found, they were considered as factors that could reduce the negative outcomes.

While the incidence of irAEs is an unfavorable outcome available from case reports, it was difficult to evaluate because we could not make comparisons. We have provided the incidence rates of irAEs in renal transplant recipients with those in the non-recipients as reference information, for negative outcomes.

There were no significant differences in the response rate to ICIs and incidence rate of irAEs in patients who have undergone renal transplantation compared with the general population without renal replacement therapy; therefore, the use of ICIs was evaluated as not harmful in patients who have undergone renal transplantation.

## Summary of evidence for CQ

[Certainty of evidence: C (weak)].

## Evaluation items to determine the strength of the recommendation

1. Factors that influence the determination of the strength of the recommendation

**There is strong overall evidence for the overall outcome [Assessment: No]**

The OS, response rate, and incidence rate of organ rejection in patients after renal transplantation were evaluated based on one observational study and accumulated case reports, and the certainty of evidence was weak.

**The benefit-harm balance is certain [Assessment: No]**

OS prolongation has been reported as a beneficial outcome, while increased risk of kidney rejection and graft loss has been reported as a detrimental outcome in patients after renal transplantation. However, as kidney rejection may be suppressed by the use of combination of multiple immunosuppressive drugs, including mTOR inhibitors, we determined that the benefits outweigh the harmful effects, although the certainty of evidence was weak.

2. Factors to consider for the strength of the recommendation

ICIs may prolong survival in patients after renal transplantation, although it also increases the risk of kidney rejection and graft loss. When graft loss occurs due to rejection, re-transplantation, or hemodialysis is considered as the consequent renal replacement therapeutic option. Re-transplantation is not indicated for patients with cancer; these patients would be indicated for re-induction of hemodialysis or peritoneal dialysis. Induction of dialysis makes it possible to continue cancer chemotherapy, although it may cause more physical and mental burden than renal engraftment. Perception of the risk of dialysis re-induction is greatly influenced by patients’ values. Additionally, ICIs are expensive, and the patient’s perception of survival benefits relative to the drug cost depend on patients’ ethics.

## Commentary on recommendation

## Background and purpose

Among the renal replacement therapies available, renal transplantation is the best treatment option when considering improvement in vital prognostic factors, improving QOL, and lower medical costs. However, oral administration of immunosuppressive drugs is essential to suppress rejection mediated by humoral and cellular immune responses that occur when a non-autologous kidney enters the body.

Patients who have undergone renal transplantation have a higher incidence of certain types of malignancies than the general population. The reasons for this include risk factors specific to patients with CKD and those due to immunosuppressive drug use, in addition to risk factors similar to those in the general population (see General review 1–3: “2. Patients who have undergone renal transplantation” [1, 52]). In particular, the activation of cancer-causing viruses and suppression of immune surveillance in cancerous cells by immunosuppressive drugs greatly contribute to the development of malignant tumors. Cancers such as non-melanoma skin cancer, melanoma, post-transplant lymphoproliferative disease, renal/urinary tract cancer, gastrointestinal cancer, and lung cancer have been reported to have high SIRs [53], although there are large racial differences, with Asians having lower incidence of skin cancer than Caucasians. However, there are reports from Japan that non-melanoma skin cancer is by no means rare [54] and that the associated SIR is higher than that of the general population because of the small number of incidence [55]. Therefore, it is considered to be a type of cancer that requires caution in Japan as well.

Under these circumstances, in addition to skin cancer (melanoma), lung cancer, renal cell carcinoma, gastrointestinal cancer, and urothelial cancer, where an ICI is indicated even for renal transplant recipients, the use of ICIs is expected to increase for other types of cancers including non-melanoma skin cancer, for which clinical trials are underway. Therefore, this CQ will become increasingly important in clinical practice.

## Target

As a result of a SR on this CQ, we found 1 report of an observational study, 48 case reports on renal transplant recipients receiving ICI therapies, and 13 reviews based on these case reports.

## Survival period

Prolonged survival is the most desirable outcome for ICI therapy. There is only one observational study that compares the survival prognosis of patients who underwent renal transplantation and received ICI therapy to that of patients who received other treatments [56]. This multicenter observational trial found that while ICI therapy did not outperform other therapies for patients with melanoma, it significantly prolonged the OS in patients with non-melanoma squamous cell carcinoma compared to those who received other therapy [56]. The same study also demonstrates that the rejection, transition to hemodialysis, and rejection at an early stage did not affect OS. A limitation of this study was that the demographics and treatment details of patients undergoing non-ICI therapy were heterogeneous and that it did not specify the drugs used by those patients. Additionally, because so few case reports collected through the SR provided information on survival, the cumulative case reports could not be used to analyze survival outcomes.

Thus, we believe that for the treatment of non-melanoma skin cancer, which has a high incidence in patients who have undergone renal transplantation, choosing ICIs is beneficial for OS, although the certainty of this suggestion is weak.

## Response rate

The response rate is an important tumor-related outcome. The observational study mentioned previously noted that the response rate for patients with non-melanoma squamous cell carcinoma treated with ICIs was higher (33.3%), than that for patients treated with non-ICIs (8.6%), and similar trends were observed in patients with melanoma [56]. According to the calculations by this CQ study group based on the 47 case reports and 101 patients found by SR (48 with melanoma, 27 with non-melanoma cutaneous squamous cell carcinoma, 8 with lung cancer, and 18 with other cancers), the response rate to ICIs was 35.3% for patients with non-melanoma cutaneous squamous cell carcinoma and 54.5% for those with melanoma, similar to the response rates shown in the observational study mentioned above. Although as uncertain as survival, the response rate may also benefit from the choice of ICI therapy for skin cancers, including melanoma.

## Risk of rejection

ICIs exert antitumor effect by activating T-cell immunity, although this activation may induce organ rejection in transplant recipients. The observational study mentioned previously reported a 42% rejection rate with usage of ICIs in patients who had undergone renal transplantation; of which, 65.5% ultimately lost the transplanted kidney [56]. In the same study, the incidence rate of rejection was 5.4% in renal transplant recipients who received non-ICI therapy, indicating that organ rejection is more likely to occur in transplant recipients using ICIs [56]. The incidence ratio of organ rejection, calculated by this CQ study group based on the 101 case reports found by SR, is 45.5%, which is comparable to the value reported in the observational study. These results suggest that renal transplant recipients using ICIs have an increased risk of organ rejection compared to those not using ICI; therefore, more careful monitoring is required.

According to the observational study stated above, using mTOR inhibitors in conjunction with immunosuppressants significantly prolongs graft survival during ICI therapy and reduces the risk of rejection [56]. Although the detailed mechanism of the protective effect of mTOR inhibitors on the transplanted kidney has not been elucidated, its influence as a secondary preventive of skin malignancy in renal transplant recipients has been shown [57], making them important drugs for suppressing organ rejection as well.

Additionally, the antitumor effect of ICI and the risk of rejection are considered to be a trade-off, and the incidence of rejection tends to be higher in patients using fewer immunosuppressive drugs at the time of ICI therapy. In the observational study mentioned above, 20% of patients without kidney rejection during ICI therapy had reduced the number of immunosuppressant drugs to 0–1, while 44.8% of patients with rejection had reduced immunosuppressant drugs to 0–1. Albeit with no significant difference, the number of immunosuppressant drugs tended to be fewer among patients with rejection. Similarly, in the accumulation of the case reports collected by this CQ study group, kidney rejection occurred in 29.4% of patients who used three immunosuppressants, in 35.4% who used two immunosuppressants, in 61.3% who used one immunosuppressant, and in all patients who did not use an immunosuppressant; thus, the incidence of kidney rejection tended to be higher in patients using fewer immunosuppressants.

The use of immunosuppressants is known to be an adverse prognostic factor in patients with cancer, and the number of immunosuppressants used by a renal transplant recipient is at times reduced following a cancer diagnosis to avoid exacerbation (see General Review 1–3: “2. patients who have undergone renal transplantation” [1, 52]). While it may be necessary to consider the use of a triple immunosuppressant regimen when initiating ICI therapy, there are no reports available to evaluate the evidence.

Thus, the observational study and case reports accumulated through our SR suggested that the risk of rejection may be reduced by continuing the concurrent use of multiple immunosuppressants, including mTOR inhibitors, at least when using ICIs.

Re-transplantation would not be an option for cancer patients if the transplanted kidney was lost; instead, hemodialysis or peritoneal dialysis would be recommended. Dialysis induction would allow cancer chemotherapy to continue, although it may cause more physical and mental burden than renal engraftment. The patient’s view of the risk of dialysis re-induction is greatly influenced by the patient’s values, and should be explained as the risks associated with ICI therapy to patients.

Although ICI therapy increases the risk of rejection in renal transplant recipients, multi-drug immunosuppressive therapy, including mTOR inhibitors, may help avoid rejection; as there are ways to continue the cancer chemotherapy by renal replacement therapy, the high risk of organ rejection does not outweigh the enormous benefits of ICI use.

## irAEs

The incidence of irAEs in patients treated with ICI therapy is a notable harmful outcome. In the observational study mentioned above, although only extrarenal lesions were evaluated, 24.6% of patients had at least one irAE. IrAEs are ICI-specific AEs and cannot be compared with those associated with non-ICI therapy. Considering the incidence of irAEs in non-recipients of renal transplant, 85% of patients with melanoma treated with ipilimumab alone reportedly developed some type of irAE [58], suggesting that the incidence of irAEs reported in renal transplant recipients is not extremely high.

## Conclusions and challenges

Thus, in this CQ, since the risk of rejection (harmful consequence of ICI) is not considered to outweigh the prolongation of OS and high response rate (beneficial outcomes of ICI), we recommended ICI therapy for renal transplant recipients.

All the studies in the SR of this CQ were case reports, except for one observational study. Therefore, it is important to note that there may be many biases. Future research must examine the survival prognosis for malignancies other than skin cancers, including melanoma, when treated with ICI vs non-ICI. Additionally, by accumulating evidence of rejection, such as studies to verify the regimen of immunosuppressive drugs to suppress rejection in renal transplant recipients using ICIs and a comparative study to verify the graft-protective effect of mTOR inhibitors, it would be possible to establish a system for managing kidney transplant rejections during ICI therapy. Furthermore, by analyzing irAEs, which are common in transplant recipients, it would be possible to identify clinical and laboratory findings that need to be monitored for better safety and to build a close cooperation system with medical departments related to the irAEs.

## CQ6: What is the recommended method of hydration to alleviate renal dysfunction during cisplatin administration in adults?

The basic approach to alleviate renal dysfunction during cisplatin treatments is to provide the patient with 1000–2000 mL of fluids over more than four hours before and after cisplatin administration. However, only for patients with good overall health and organ function who can tolerate brief hydration, short hydration is weakly recommended. After administering cisplatin, the short hydration method, which replenishes a smaller volume of fluids over a shorter period of time than conventional hydration, is only considered in facilities that provide suitable treatment environment for patients requiring additional fluid due to gastrointestinal disorders, and are equipped to handle emergencies. There are no studies providing evidence for the appropriate amount of hydration needed when administering low-dose cisplatin (< 50 mg/m^2^); thus, there is insufficient clarity about this.

Recommendation grade: Short hydration method is weakly recommended (proposed) (agreement rate: 96.3%, votes: 27 voters, agreement: 26 voters).

Values and preferences associated with recommendations (Assuming a set of values for each outcome considered)

As a recommended hydration method to mitigate renal dysfunction during cisplatin administration, a conventional hydration method is used in Japan in accordance with package instructions of the cisplatin injection/IV drip infusion [59, 60]. It is difficult to evaluate the evidence on the need for hydration during cisplatin administration because there is no clinical trial comparing cisplatin treatments with and without hydration. Therefore, in this CQ, we continued to use the conventional hydration method as the recommended method to mitigate renal dysfunction during cisplatin administration.

A SR was conducted on the short hydration method, which replenishes smaller volumes of fluids over a shorter period of time than conventional hydration. The overall assessment of the evidence was a grade of C (weak), which is the same as the recommendation grade assigned in the 2016 edition of these guidelines. A SR showed that approximately 20% of cases who were under the short hydration method, required additional hydration due to gastrointestinal disorders after administration of cisplatin [61–65]. The decision to perform short hydration in a patient with good overall health and organ function, that can withstand short hydration, should be taken after discussions with the patient, considering the patient’s values and preferences and circumstances of the institution.

We conducted a SR on the appropriate amount of hydration for low-dose cisplatin administration; however, there were no papers available to evaluate the evidence, and it could not be examined directly.

## Summary of evidence for CQ

(Certainty of evidence: C [weak]).

## Evaluation items to determine the strength of the recommendation

1. Factors that influence the determination of the strength of the recommendation

**There is strong overall evidence for the overall outcome [Assessment: No]**

All five selected papers [61–65] reported single-arm interventional studies evaluating the safety of short hydration in high-dose cisplatin treatments on small-scale, and there were no reports on RCTs (certainty of evidence: C [weak]).

**The benefit-harm balance is certain [Assessment: Yes]**

There were no papers providing evidence on OS, PFS, and response ratio, although there were reports indicating that short hydration is beneficial for avoiding inpatient treatment (certainty of evidence: C [weak]). There were no papers available to evaluate the evidence on QOL and patient satisfaction.

2. Factors to consider for the strength of the recommendation

Approximately 20% of patients who use the short hydration method after administration of cisplatin are expected to require additional hydration due to gastrointestinal disorders. It is necessary to consult with the patient in advance to decide whether to use conventional hydration or short hydration, depending on the patient’s values, preferences, and circumstances of the institution.

## Commentary on recommendation

## Background and purpose

Preclinical studies (animal experiments) identified cisplatin as being nephrotoxic. To alleviate renal dysfunction due to tubular injury during cisplatin administration, it is important to ensure appropriate urine output and to promote smooth excretion of free cisplatin. Thus, in Japan, it is conventional to provide patients with 1000–2000 mL fluids for at least 4 h before and after cisplatin administration and to administer cisplatin diluted with at least 500–1000 mL fluids for at least 2 h (a total of 2.5–5 L before, during, and after cisplatin administration [conventional hydration]) [59, 60]. In this CQ, we continued to adopt the conventional hydration method, which has been used as the recommended hydration method to alleviate renal dysfunction during cisplatin administration; we conducted a SR on the short-hydration method, which replenishes smaller volumes of fluids over a shorter period of time (a total of 1,600–2,500 mL of fluids given intravenously before and after cisplatin administration and approximately 1000 mL of water given orally on the same day, by the end of cisplatin administration. See General review 9 in Chapter 3 Clinical Practice Guidelines for the Management of Kidney Injury During Anticancer Drug Therapy 2022 [1, 66]). Cisplatin treatment regimens are classified as high dose (≥ 50 mg/m^2^) and low dose (< 50 mg/m^2^) depending on the dose administered. We also conducted a literature search on the appropriate amount of replacement fluid to be used during low-dose cisplatin treatments.

## Target

After searching through the literature using cisplatin, hydration, nephrotoxicity, and magnesium as keywords, our primary screening detected 461 hits on PubMed®, 48 on Cochrane Library, and 53 on the ICHUSHI-web databases (total of 562 hits). Five papers [61–65] on short hydration during cisplatin administration were extracted through the secondary screening, and a qualitative SR was conducted. All five reports were on small, single-arm interventional studies evaluating the safety of short hydration in high-dose cisplatin administration and were not RCTs.

## Safety assessment

In all five reports, the incidence ratios for renal dysfunction and the requirement for additional hydration due to gastrointestinal disorders associated with administration of cisplatin in low hydration were 3.6 and 19.4%, respectively.

Based on the above, the overall assessment of the evidence was grade C (weak), similar to the grade recommended in the 2016 edition of these guidelines [67], and the short hydration method during cisplatin administration was weakly recommended for hydration to mitigate renal dysfunction. Notably, approximately 20% of cases required additional hydration due to gastrointestinal disorders. Therefore, the usage of the conventional or short-hydration procedure should be decided carefully, as described above.

There was only one report [64] providing evidence on the efficacy and safety of oral hydration solutions (OS-1®, among others) after cisplatin administration as part of the short hydration method; this should be examined in future. Due to inconsistent patient statuses (type of cancer and setting) and histological classification of cancer during cisplatin administration, patient survival and the response rate could not be evaluated.

## Fluid volume during low-dose cisplatin treatments

Despite conducting a SR on the appropriate fluid volume to be used during low-dose cisplatin (< 50 mg/m^2^) treatment, there were no published reports available on its evidence; thus, we could not study it directly. In an RCT [68] that verified the efficacy and safety of gemcitabine + cisplatin combination therapy (cisplatin 25 mg/m^2^), which is the standard of care for biliary tract cancer, patients were administered 1000 mL hydration during cisplatin and 500 mL hydration during gemcitabine administration as outpatient treatments. This may be a reference hydration method for low-dose cisplatin treatment, although the appropriate fluid volume during low-dose cisplatin treatment remains unknown as there are no other papers providing evidence on this.

## CQ7: Is the use of anti-angiogenic agents recommended for patients with proteinuria or a history of proteinuria?

Even though there is weak evidence that the presence of proteinuria at the start of anti-angiogenic agent administration is a risk factor for proteinuria exacerbation, anti-angiogenic agents can be administered to patients with or without proteinuria because there is no significant association with more important outcomes such as mortality or decline in the eGFR.

**Recommendation grade**: Weakly recommended (proposed) (agreement rate: 100%, votes: 28 voters, agreement: 28 voters).

Values and preferences associated with recommendations (Assuming a set of values for each outcome considered)

Considering the recommendations for this CQ, we focused on whether administration of anti-angiogenic agents to patients with a history of proteinuria would lead to serious side effects.

## Summary of evidence for CQ

(Certainty of evidence: D [very weak]).

## Evaluation items to determine the strength of the recommendation

1. Factors that influence the determination of the strength of the recommendation

**There is strong overall evidence for the overall outcome [Assessment: No]**

The five selected papers are all cohort studies.

**The benefit-harm balance is certain [Assessment: No]**

Although a history of proteinuria is a significant risk factor for exacerbation of the condition after administration of anti-angiogenic agents, there is no study on the association between a history of proteinuria and deterioration of the eGFR or the development of nephrotic syndrome.

2. Factors to consider for the strength of the recommendation

Patient (family) preferences for this treatment may vary widely. As patients discontinue or reduce the dose of molecular targeted therapies for the reason of disease aggravation, it is unclear whether this will lead to reduction in overall costs.

## Commentary on recommendation

## Summary

Although there are papers providing evidence that the presence of proteinuria at the start of administration of anti-angiogenic agents is a risk factor for proteinuria exacerbation, it has no significant association between the presence of proteinuria and the deterioration of renal function, suggesting that it is possible to administer anti-angiogenic agents irrespective of the presence of proteinuria.

## Background and purpose

Anti-angiogenic agents which are small molecule compounds or antibody drugs, are used to treat various cancers. Proteinuria is a typical side effect of anti-angiogenic agents, sometimes leading to renal impairment and nephrotic syndrome. Therefore, in this section, we examined whether anti-angiogenic agents can be administered safely to patients with proteinuria or a history of proteinuria.

## Target

For CQ7, we conducted a literature search focusing on whether the administration of anti-angiogenic agents to patients with a history of proteinuria or those co-developing proteinuria would lead to serious side effects. The participants were patients treated with anti-angiogenic agents. The treatment-exposed arm was grade 1 or higher proteinuria-positive at the start of treatment, while the control group was proteinuria-negative at the start of treatment. Mortality, decrease in the eGFR, and progression of proteinuria were set as outcomes. We extracted five retrospective observational studies [69–73]. All of these studies were on patients with renal cell carcinoma. In these observational studies, the majority of proteinuria-positive patients had proteinuria 1 + or < 1 g/day, as many patients with proteinuria ≥ 2 + or ≥ 1 g/day (g/gCr) tend to avoid treatment.

## Mortality

Mortality was reported in a study of 45 cases in Japan. In a multivariate Cox proportional hazard model with mortality as an outcome, the hazard ratio for baseline proteinuria (1 + or higher) was 0.82 (95% CI 0.23–2.97), with no significant association with mortality [69]. Additionally, since being proteinuria-positive at the beginning of anti-angiogenic agent treatment was not a major exposure, there were no differences in baseline characteristics between proteinuria-positive and proteinuria-negative patients.

## Decrease in the eGFR

A report that evaluated the changes in the eGFR before and after anti-angiogenic drug treatment in 41 patients who had proteinuria at the start of treatment was published [70]. Although this study found no significant deterioration in renal function, the proteinuria-positive population in this study included 27 patients who developed the condition after the study’s treatment began and 14 patients who were already positive at baseline. The analysis only for the patients who were already proteinuria-positive at baseline was not performed. Additionally, this trial had only one arm, and it compared patients before and after therapy, not patients with and without proteinuria. The study was limited to patients who received treatment for ≥ 12 weeks; thus, patients who terminated the study early due to renal impairment as an AE may have been selectively excluded.

## Proteinuria

There were three reports on proteinuria, and in the two reports where data on proteinuria at baseline were collected, each was defined as having proteinuria of 2 g/day or more or proteinuria of any grade. Despite differences in criteria, the results of the reports were consistent because proteinuria at baseline is a significant risk factor for proteinuria exacerbation after the start of the treatment [71, 72]. One report lacking information about baseline proteinuria showed that most cases of proteinuria were of grade 1 or 2; treatment using anti-angiogenic agents could continue without dose reduction or treatment interruption [73].

After starting the administration of anti-angiogenic agents, it is necessary to monitor proteinuria appropriately and carefully judge the benefits and harmful effects of continuing treatment with anti-angiogenic agents according to the grade of proteinuria.

## CQ8: Is additional magnesium supplementation recommended if a patient receiving anti-Epidermal Growth Factor Receptor (EGFR) antibody develops hypomagnesemia?

Additional magnesium supplementation is weakly recommended when a patient receiving anti-EGFR antibody develops hypomagnesemia, as additional magnesium supplementation may help avoid aggravation of hypomagnesemia.

**Recommendation grade**: Weakly recommended (proposed) (agreement rate: 100%, votes: 28 voters, agreement: 28 voters).

Values and preferences associated with recommendations (Assuming a set of values for each outcome considered)

In preparing the recommendations for this CQ, we focused on suppressing the exacerbation of hypomagnesemia at the onset of hypomagnesemia during anti-EGFR antibody therapy.

## Summary of evidence for CQ

(Certainty of evidence: D [very weak]).

## Evaluation items to determine the strength of the recommendation

1. Factors that influence the determination of the strength of the recommendation

**There is strong overall evidence for the overall outcome [Assessment: No]**

The strength of the evidence is D.

**The benefit-harm balance is certain [Assessment: No]**

Although progression of Common Terminology Criteria for Adverse Events (CTCAE) grade 1 hypomagnesemia to grade 2 hypomagnesemia could be suppressed in some patients receiving intravenous magnesium infusion, there is no study whether patients could continue to receive anti-EGFR antibody with the additional magnesium infusion.

2. Factors to consider for the strength of the recommendation

Patient (family) preferences for this treatment may vary widely. Drug costs (unit cost) are relatively cheap, although it is unclear how entire costs during anti-EGFR antibody therapy can be reduced if aggravation of hypomagnesemia is suppressed.

## Commentary on recommendation

## Summary

For hypomagnesemia developing after administration of anti-EGFR antibody, additional magnesium supplementation has no apparent harm, and it may help avoid aggravation of hypomagnesemia.

## Background and purpose

Anti-EGFR antibody, such as cetuximab and panitumumab, are used either alone or in combination with other anticancer agents as a standard treatment for RAS wild-type colorectal cancer, head and neck cancer, and squamous cell lung carcinoma. When anti-EGFR antibody is used in pharmacotherapy, hypomagnesemia is an adverse effect that is relatively common; it should be treated with caution since when it is severe, it can lead to arrhythmia. Although serum magnesium monitoring is a common approach, there is no established standard on magnesium supplementation for hypomagnesemia. We examined the standards for magnesium supplementation, particularly as a countermeasure against hypomagnesemia caused by anti-EGFR antibody.

## Target

For this CQ8, we searched for the literature, emphasizing suppression of hypomagnesemia exacerbation at hypomagnesemia onset during administration of anti-EGFR antibody drugs. There were no studies involving a control group, and finally, we extracted two case series studies and one SR [74–76].

## Results

Demizu et al. [74] formulated an in-hospital manual indicating “to start oral magnesium oxide from the start of cetuximab treatments and, in patients who develop hypomagnesemia, to start intravenous (IV) infusion of magnesium sulfate from the onset of point of grade 1 (CTCAE) hypomagnesemia” for patients receiving cetuximab, an anti-EGFR antibody. Consequently, while hypomagnesemia occurred in 9 of 10 patients after cetuximab treatments before the formulation of the in-hospital manual (grade 1: 7 patients; grade 2: 1 patient; and grade 3: 1 patient), 4 of 5 developed grade 1 hypomagnesemia after the formulation of the in-hospital manual with no deterioration to a grade 2 or higher grade. This interventional study suggested that IV infusion of magnesium from the onset of grade 1 hypomagnesemia may help avoid the aggravation of hypomagnesemia.

However, there is no information in the literature regarding whether it was possible to continue the anti-EGFR antibody treatment or whether the treatment was interrupted or postponed after the onset of hypomagnesemia. Additionally, no studies have examined whether treating hypomagnesemia could prevent the development of arrhythmias as a clinical consequence.

Although there is no report on the obvious harmful effects from magnesium supplementation, we currently await the accumulation of cases as there is no large-scale study available.

## CQ9: Is it recommended to discontinue steroids being used to treat kidney injuries caused by ICIs after normalization of renal function?

When kidney injuries caused by the use of ICIs are treated with steroids, the usefulness of continued administration of steroids after normalization of renal function is unclear as there are concerns about the increase in AEs and attenuated therapeutic effects of ICIs. It is weakly recommended to discontinue steroid after carefully considering the risk of kidney injury recurrence following treatment discontinuation and the measures to be taken in the event of a recurrence.

**Recommendation grade**: Weakly recommended (proposed) (agreement rate: 100%, votes: 27 voters, agreement: 27 voters).

Values and preferences associated with recommendations (Assuming a set of values for each outcome considered)

Important outcomes included treatment delays or interruptions (importance: 8 points), response rate, grade 3 or higher of elevated serum Cr levels (importance: 6 points), and recurrence of kidney injuries (importance: 5 points).

## Summary of evidence for CQ

[Certainty of evidence: D (very weak)].

## Evaluation items to determine the strength of the recommendation

1. Factors that influence the determination of the strength of the recommendation

**There is strong overall evidence for the overall outcome [Assessment: No]**

Three papers extracted from the literature search were case series with a limited number of cases. It was difficult to evaluate whether treatment was interrupted or delayed because it was interrupted in all patients. One paper reported on response rates and grade 3 or higher of elevated serum Cr levels, defined as an increase in SCr > threefold above baseline, or an increase in SCr to a level > 4.0 mg/dL. Both the response rate and increased serum Cr level were difficult to evaluate due to the very small number of cases. The strength of evidence was deemed very weak (D).

**The benefit-harm balance is certain [Assessment: No]**

The therapeutic effect of ICIs may be weakened by steroids. A poor prognosis has been reported in patients treated with steroids for the palliation of cancer-related symptoms, although it is believed that this is due to the subjects being in the population with worst disease condition. There is not enough evidence to suggest that the use of steroids, when not associated with relieving cancer symptoms, does not affect the therapeutic efficacy of ICIs.

2. Factors to consider for the strength of the recommendation

The risks of various AEs such as infection, osteoporosis, disorders of carbohydrate metabolism, weight gain, edema, and eye diseases occur with continued steroid treatments. The cost of testing, treatment, and hospital visits for managing AEs may also increase.

## Commentary on recommendation

## Summary

If kidney injuries caused by ICI use are treated with steroids, should steroids be discontinued after renal function is normalized? In response to this question, we weakly recommend stopping steroid treatment after considering the risk of recurring kidney injuries sufficiently, following treatment suspension and in case of recurrence, as the usefulness of continued steroid administration is not clear and there are concerns about an increase in AEs and attenuated therapeutic efficacy of ICIs due to continued treatment.

## Background and purpose

ICIs may cause a variety of irAEs. Guidelines have been issued by the American Society of Clinical Oncology (ASCO) regarding response to various irAEs, and diagnosis and treatment are conducted in accordance with these guidelines [77]. The guidelines also include recommendations on kidney injuries according to their severity. Specifically, the guidelines recommend interrupting ICI treatments and administering steroids for grade 2 or higher renal dysfunction. After steroid therapy induction, the dose should be tapered over ≥ 4 weeks while checking for the improvement of the renal function. This is a crucial clinical issue because there is no clear advice regarding whether steroid therapies should continue after renal function has returned to normal based on the positive and negative results, such as irAE suppression and the impact on ICI efficacy.

We aimed to examine the latest findings on the beneficial and harmful effects of continuing steroid therapy after renal function has normalized when kidney injuries caused by ICI use are treated with steroids, and to elucidate its usefulness and limitations in practical clinical use.

## Target

Following literature search for this CQ, we extracted 214 reports subject to screening, including 154 PubMed, 5 Cochrane, 54 Japan Medical Abstracts Society reports, and an additional record identified by hand-searching. Qualitative evidence assessment was conducted on three reports extracted following two rounds of screening. All three reports were case series with a limited number of cases. Important outcomes included treatment delays or interruptions, response rate, grade 3 or higher elevated serum Cr level, and recurrence of kidney injuries. A meta-analysis was not conducted considering the number of cases, outcomes, and similarity to PICO.

## Response rate and elevated serum Cr level

One of the above-mentioned reports evaluated the response rate and increased serum Cr level, and the response rates of the steroid-continuation and steroid-discontinuation arms were 100% (2/2 cases) and 64% (7/11 cases), respectively. Grade 3 or higher serum Cr elevation was observed in 100% (2/2 cases) and 82% (9/11 cases) of patients, respectively [78]. However, other literature did not mention these outcomes, making comparative verification difficult. Although we examined treatment delays or interruptions as another important outcome, it was not possible to compare them between the two arms since steroid treatments were suspended or discontinued in all patients.

Based on these results, there was very weak evidence (D) about whether steroid treatments should be continued at a tapered dose even after normalization of renal function.

## Impact on therapeutic effect

There are several reports investigating the impact of steroid administration on the therapeutic effect of ICIs. According to a study of two institutions that examined the clinical and drug dispensing records of 640 patients with advanced non-small cell lung cancer treated with a programmed death-ligand 1 (PD-(L)1) inhibitor alone, those who received a prednisolone (PSL) equivalent dose of ≥ 10 mg/day (90 patients, 14.1%) at the start of PD-(L)1 inhibitor treatment had a poor overall response rate, PFS, and OS. PFS (hazard ratio 1.3, *p* = 0.03) and OS (hazard ratio 1.7, *p* < 0.001) were poor even after adjusting for smoking history, performance status, and brain metastasis by multivariate analysis [79]. Thus, steroid administration was suggested to attenuate the therapeutic effect of ICIs.

## Steroid use for palliation of cancer-related symptoms

There has been a report indicating that steroids not associated with palliation of cancer-related symptoms may not reduce the effectiveness of ICIs. In a single-center study of 650 patients with advanced non-small cell lung cancer treated with ICIs, those who received a PSL-equivalent dose of ≥ 10 mg/day at the start of treatment (93 patients, 14.3%) and those who received a dose of 0–10 mg/day (557 patients, 85.7%) were compared, and the former had a shorter median PFS (mPFS) and OS (mOS) (mPFS 2.0 vs. 3.4 months, *p* = 0.01; mOS 4.9 vs. 11.2 months, *p* < 0.001).

The group that received a PSL dose of ≥ 10 mg/day for causes unrelated to cancer symptom alleviation showed no significant differences in the mPFS and mOS compared with the group that received a PSL dose of 0–10 mg/day. The mPFS and mOS were poor only in the group that received a PSL dose of ≥ 10 mg/day for relieving cancer symptoms. This was because this subgroup showed a poor disease prognosis. The use of PSL unrelated to palliation of cancer symptoms may not attenuate the therapeutic effect of ICIs, although it is not clear whether the therapeutic effect of ICIs remains unaffected even if the steroid dose is increased to ≥ 10 mg/day [80]. Notably, this study is subject to various biases, such as the small number of patients who received steroid therapy for purposes other than symptom relief; moreover, this was a single-center, retrospective study. It is also unclear whether similar results can be obtained for cancers other than non-small cell lung cancer.

## Adverse events due to steroid drugs

An increase in AEs associated with steroid administration need to be considered. Patients treated with steroids could be at increased risk of various AEs such as osteoporosis, weight gain, disorder of carbohydrate metabolism, infections, edema, mood disorders, and eye diseases [81, 82].

## Conclusion

As mentioned above, the effect of continued steroid therapy after normalization of renal function on the therapeutic effect of ICIs and risk of recurrence of kidney injuries is unclear at present. However, there are concerns that continued administration of steroids may increase the risk of AEs, medical costs associated with their evaluation and management, and frequency of medical examinations, which may become a burden on patients. Thus, we decided to recommend terminating the administration of steroids weakly, after thorough discussions between the oncologist and nephrologist regarding the risk of recurrence of kidney injuries after stopping steroid treatment and taking measures against recurrence.

## CQ10: Is the resumption of ICI recommended after a patient has recovered from kidney injuries associated with ICIs?

Despite concerns about the recurrence of associated kidney injuries when ICI treatment is resumed in a patient who have recovered from kidney injuries, it is weakly recommended to resume the treatment if its merits outweigh the demerits.

**Recommendation grade**: Weakly recommended (proposed) (agreement rate: 100%, votes: 27 voters, agreement: 27 voters).

Values and preferences associated with recommendations (Assuming a set of values for each outcome considered)

Outcomes included treatment delays or interruptions, response rate, grade 3 or higher serum Cr elevation, and recurrence of kidney injuries. Among them, treatment delays or interruptions was considered an important outcome (importance: 8 points), followed by response rate, and ≥ grade 3 serum Cr elevation (importance: 6 points).

## Summary of evidence for CQ

(Certainty of evidence: D [very weak]).

## Evaluation items to determine the strength of the recommendation

1. Factors that influence the determination of the strength of the recommendation

**There is strong overall evidence for the overall outcome [Assessment: No]**

The seven papers extracted by literature search were all case series with a limited number of cases. There was no mention of control groups for each outcome; therefore, evaluation by comparison with the intervention groups was impossible. The data on the benefits and harmful effects of the intervention group in the individual papers were suggestive, although the risk bias was significant, and the evidence was very weak (D).

**The benefit-harm balance is certain [Assessment: No]**

In terms of the effectiveness (benefit) of ICIs, the type of cancer, biomarkers (PD-L1 expression, microsatellite instability [MSI], and tumor mutational burden [TMB] [see commentary on recommendation]), and therapeutic effect during ICI administration are reference indicators. Considering harmful effects, the recurrence rate of kidney injury after ICI re-administration varied widely in the articles extracted from the literature search. The degree of risk of kidney injury recurrence upon re-administration is not clear. It is difficult to draw a certain conclusion about the balance between benefits and harmful effects, and careful consideration is needed case-by-case.

2. Factors to consider for the strength of the recommendation

Re-administration of ICIs may increase the cost of treatment and burden of hospital visits. It is also necessary to consider the incidence and severity prediction of recurrence and new onset of irAEs, including kidney injury, and the availability, efficacy, and safety of other treatment options.

## Commentary on recommendation

## Summary

We weakly recommend the re-administration of ICIs after a patient recovers from ICI-associated kidney injuries when the merits outweigh their demerits, considering the risk of kidney injury recurrence, possibility of other irAEs, type of cancer being treated, past treatment outcome, response-predictive biomarkers, and availability of other treatment options.

## Background and purpose

ICIs are effective against a variety of cancer types and play a crucial role in anticancer drug therapy today. It is known that irAEs, associated with reactivation of immunity occur during ICIs use. Among kidney-associated irAEs, AKI is reported to be a common clinical feature, with acute interstitial nephritis accounting for the majority of its pathology, and generally showing a favorable response to corticosteroids [78, 83].

The oncologist and nephrologist should cooperate appropriately to diagnose and to treat ICI-related kidney injuries. It is common to examine and treat these patients according to the guidelines of the ASCO and United States National Comprehensive Cancer Network. It is recommended that grade 2 (serum Cr levels elevated to two- to three-fold the baseline level) AKI is treated with corticosteroids while temporarily discontinuing ICI, the resumption of which is considered after normalization of renal functions. For grade 3 or higher AKI (serum Cr levels elevated to more than three-fold the baseline level or elevated to > 4.0 mg/dL), it is recommended that the patients be treated using steroids or immunosuppressants and permanently discontinue ICI treatment [77]. However, there are no recommendations based on sufficient evidence regarding the resumption of ICI treatment for patients whose renal functions have normalized with steroid administration, and it remains an important clinical issue. The purpose of this CQ is to examine the latest findings on the benefits and harmful effects of resuming ICI treatment after patients have recovered from kidney injuries caused by ICI treatment and to elucidate its usefulness and limitations in practical clinical use.

## Target

Following literature search for this CQ, we extracted 205 reports subject to screening, including 137 PubMed, 8 Cochrane, 59 Japan Medical Abstracts Society reports, and an additional record identified by hand-searching. Qualitative evidence assessment was conducted on seven reports extracted through two rounds of screening. All seven reports were case series with a limited number of cases [84–90]. Important outcomes were treatment delays or interruptions, response rate, grade 3 or higher serum Cr elevation, and recurrence of kidney injuries.

## Recurrence of kidney injuries

The rate of recurrence of kidney injuries upon re-administration of ICIs varies significantly from 5.1 to 100% (mean of 17.9%, 26/145 cases); moreover, there was no information on patients who did not resume ICI treatment; thus, we could not make comparisons regarding the risk of kidney injury recurrence upon re-administration of ICIs. There was also no information about outcomes related to other benefits and harmful effects in the population of patients that did not resume ICI treatment; thus, we could not make comparisons between patients who did and did not resume ICI treatment. Data on recurrence of kidney injury were suggestive, and due to high risk of bias involved, they were regarded as very weak (D) evidence.

## Response-predictive biomarkers

When considering anticancer drug therapy, it is important to clarify the treatment goals (cure, prolongation of life, and QOL improvement) and to sufficiently consider the balance of benefits and harmful effects anticipated from treatment. For determination of ICI indication, research is underway on response-predictive biomarkers to extract patients who are expected to respond to ICI. PD-L1 expression, MSI, and TMB of tumor and immune cells quantified by immunohistochemical analysis of tumor tissue are already being used. In addition to the type and progression of cancer, these biomarkers are useful for assessing the efficacy of ICIs.

PD-L1 is the most widely validated and utilized response-predictive biomarker that helps to select patients who would respond to anti-PD-1/PD-L1 antibodies. PD-L1 is expressed on immunocompetent cells and tumor cells, and by binding to PD-1 on T cells, it attenuates T-cell immune responses and induces immune tolerance. Blocking this immune escape mechanism and reactivating the antitumor immune response are the rationale for treatment with anti-PD-1/PD-L1 antibodies [91]. MSI is a phenomenon wherein microsatellite repeats exhibit a different number of repeats compared with that in normal tissues due to an impaired mismatch repair (MMR) mechanism, that repairs errors in the base sequences that occur during DNA replication during cell division. Tumors with high-prevalence MSI (MSI-high) are highly immunogenic as a result of the accumulation of gene mutations in the tumor followed by the associated production of neoantigens. TMB is defined as the number of somatic mutations per megabase of genomic sequence. The United States FDA (and subsequently Japan) approved pembrolizumab for treating solid tumors with MSI-high/MMR deficiency and high TMB, as evidence suggested that high TMB was a response-predictive biomarker of ICI use, similar to MSI-high [92, 93].

## Degree of influence of irAEs

Recommendations for ICI re-administration after improvement of irAEs vary for individual organs affected by irAEs. Basically, the permanent discontinuation of ICIs is recommended for patients presenting with grade 4 toxicities, while patients with endocrine abnormalities that can be managed with hormone replacement therapy may be able to resume using ICIs. Furthermore, besides neurological, hematological, and cardiac irAEs, which are high-risk at onset, ICI treatment may be resumed for up to grade 3 toxicities if the symptoms have alleviated to grade 1 (for kidney injuries: serum Cr level > 0.3 mg/dL or 1.5–2.0 times above baseline) or less. As mentioned above, permanent discontinuation of ICIs is recommended for grade 3 or higher kidney injuries [77]. However, when an ICI is strongly anticipated to be effective, it could be re-administered considering the possibility of recurrence of kidney injuries leading to loss of renal function and initiation of dialysis.

## Conclusion

As described above, re-administration of ICIs after a patient has recovered from kidney injuries requires consideration of various factors such as the risk of irAEs, including recurrence of kidney injuries, type of cancer and condition of cancer, response-predictive biomarkers of ICI, past treatment outcome, availability of other treatment options, and patient preferences; thus, it would be difficult to provide a uniform recommendation on the matter. Based on the above, we decided to recommend ICI re-administration to patients weakly based on close cooperation with the treating oncologist and nephrologist and when the merits of ICI treatment outweigh its demerits.

## CQ11: Is the administration of erythropoiesis-stimulating agents (ESAs) recommended for renal anemia in cancer survivors?

Treatment with ESAs for severe renal anemia is expected to reduce the amount of blood transfusion and iron replacement in patients with CKD who are in the pre-dialysis or dialysis stage. On the other hand, in pre-dialysis CKD patients with a history of cancer, ESA treatment for renal anemia was reported to increase cancer mortality when the target hemoglobin (Hb) level was high. Therefore, we concluded no recommendation on this clinical question. ESA treatment for severe renal anemia should be performed based on careful consideration of possible benefits and harmful effects, and when started, patients should be carefully monitored for cancer development.

**Recommendation grade**: Not graded (see the commentary on recommendation for the agreement rate).

Values and preferences associated with recommendations (Assuming a set of values for each outcome considered)

There are no prospective interventional studies or cohort studies specifically examining the validity of treatment with ESAs for renal anemia in CKD patients with a history of cancer. In a prospective study of renal anemia in pre-dialysis CKD patients treated with ESAs, there was a sub-analysis including patients with a history of cancer, and the SR suggested the possibility of an increase of cancer mortality. On the other hand, this report did not mention beneficial effects, such as improvement in Hb level or QOL, in this subgroup. And their targeted Hb level was higher than the recommended Hb level in the current guidelines (Hb level > 13 g/dL). Because only one report was included in the SR, there may be serious selection bias and indirectness in this analysis.

Based on this result, the team in charge of preparing a recommendation for this CQ initially submitted a draft to the panel committee that ESA treatment cannot be recommended for pre-dialysis CKD patients with a history of cancer with low certainty of the evidence.

However, at the first panel meeting (December 19, 2021), this draft was considered to be inappropriate based on the limited information from a single sub-analysis study, considering that CKD patients with a history of cancer could benefit from ESA treatment (such as improvement of levels of Hb and QOLs). After a re-assessment by the task team, it was finally concluded that the panel committee would label this topic as “not graded”, because in addition to the selection bias and indirectness of the paper included in the SR, there were concerns about the harmful effects of untreated severe renal anemia. It was also concluded that ESA treatment should be considered on a case-by-case basis by weighting the benefits and harmful effects,

The decisions should be made for individual patients by considering the background of the patient’s cancer (for instance types of tumor, clinical stage, and years after complete remission), presence of complications (such as history of thromboembolism, cardiovascular and cerebrovascular diseases, diabetes, hypertension, and dyslipidemia), living environment (like age, types of work, smoking habits, and convenience of going to hospital), and patient’s own preferences. However, when performing ESA treatment, it is necessary to prevent too much Hb elevation, to monitor cancer recurrence, and to pay attention to the development of new cancers.

## Summary of evidence for the CQ (overall strength of evidence for critical outcomes)

[Certainty of evidence: C (weak)].

## Evaluation items to determine the strength of the recommendation

1. Factors that influence the determination of the strength of the recommendation

**There is strong overall evidence for the overall outcome [Assessment: No]**

There was only one report of subgroup analysis in a prospective interventional cohort study that included target patients.

**The benefit-harm balance is certain [Assessment: No]**

The subgroup analysis reported that cancer mortality increased after ESA treatment, and it is possible that the harmful effects outweigh the benefits, although it is difficult to evaluate this as information on the benefits has not been shown.

2. Factors to consider for the strength of the recommendation

It would be inappropriate not to recommend ESA treatment based on information about some outcomes, which could be included in a SR, considering that patients with a history of cancer could benefit from ESA treatment (in terms of Hb level and QOL improvement). It was concluded that there would be "Not graded" on the matter and that ESA treatment should be considered on a case-by-case basis by weighing the benefits and harmful effects.

## Commentary on recommendation

## Background

Renal anemia is mainly caused by decreased erythropoietin production, iron deficiency, and shortened erythrocyte life span; it is known to be associated with malaise, decreased QOL, cardiovascular disease, and short life [94]. In addition to ESA administration and iron supplementation, hypoxia-inducible factor (HIF)- prolyl hydroxylase (PH) inhibitors that target HIF-PH, which regulates HIF have been used since 2019 to treat renal anemia.

Although the definition of “cancer survivors” broadly includes all patients diagnosed with cancer; this CQ concerns patients who have completed active cancer treatment (so-called "patients under observation" or considered cured). Additionally, anemia that develops during cancer treatment is cancer-and-chemotherapy-induced anemia, which is a disease concept beyond the scope of this CQ. For the diagnosis and treatment of renal anemia in patients with CKD, which is being covered in this CQ, it can be referred in the guidelines of the United States and Europe [95, 96]. A statement is also provided in the guidelines for the treatment of renal anemia in patients with CKD issued by the Japanese Society for Dialysis Therapy [97].

A SR reported that ESA therapy for renal anemia in CKD patients in the pre-dialysis or dialysis stage can reduce the amount of blood transfusion and iron supplementation, although the impact on survival and QOL is unclear [94]. Additionally, setting a high target Hb value may increase the incidence of thromboembolism such as cerebrovascular and cardiovascular diseases but may not exacerbate cancer [98]. As for QOL, Japanese guidelines recommend that treatment of renal anemia should be started when multiple episodes of severe anemia (Hb level < 10 g/dL) are observed [97]. Many guidelines recommend a target Hb value of 10–12 g/dL [95, 97, 99], while the KDIGO guideline recommends a lower target Hb level of 9.0–11.5 g/dL [100, 101].

## Systematic review

We performed a literature search to conduct a SR on this CQ. There were no prospective interventional studies that examined the suitability of ESA treatment for renal anemia in CKD patients with a history of cancer. Additionally, many cohort studies excluded cases with a history of cancer, which could not be included in this SR as it is beyond the scope.

In a prospective study of renal anemia in pre-dialysis CKD patients treated with ESAs, we could extract one subgroup analysis of patients with a history of cancer [102]. All-cause mortality and all-cancer mortality were reported as important outcomes. In spite of insufficient randomization and the inability to completely eliminate selection bias due to the subgroup analysis, all-cause mortality was 1.37 times higher (95% CI: 0.91–2.07, *p* = 0.13) and cancer mortality was 24.9 times higher (95% CI: 3.26–190.08, *p* = 0.002) in the ESA treatment group than in the non-treatment group among patients with renal anemia caused by CKD after cancer treatment. Although there was no beneficial information for this subgroup analysis and indirect biases could not be ruled out, the main analysis of this study reported that treatment with ESA improved the Functional Assessment of Cancer Therapy (FACT)-Fatigue scores.

## Review of recommendations

Based solely on the results of the SR, it was considered difficult to uniformly recommend ESA treatment to pre-dialysis CKD patients with a history of cancer. Hence, consistent with the initial recommendation, we considered that “ESA treatments are not recommended for pre-dialysis CKD patients with a history of cancer (certainty of evidence is weak).” However, this report was based on only one study, and the intervention group had a high target Hb level of 13 g/dL, which may have emphasized the mortality outcome. Additionally, even in the non-intervention group, ESA therapy was permitted as a rescue therapy when an Hb level of < 9 g/dL supported not ruling out ESA treatment for severe anemia.

At the expert panel meeting, 4 people voted in favor (22 people against, agreement rate of 15.4%) of the statement “ESA treatments are not recommended for pre-dialysis CKD patients with a history of cancer (certainty of evidence is weak).” Despite a history of cancer, there are many patients with CKD who can maintain their QOL with ESA treatment for renal anemia. Additionally, there is a lack of research on the types of cancer and patient demographics in which ESA treatment may cause cancer recurrence or development of new cancer, and there are no recommended measures. The negative aspects of not treating renal anemia adequately should also be considered. Therefore, we held a second vote on the second draft statement “ESA treatments are recommended for pre-dialysis CKD patients with a history of cancer (certainty of evidence is weak)”, and 10 people voted in favor (16 people against, agreement rate of 38.5%). This draft statement was rejected as well, and ultimately, we concluded that there is "No recommendation" for this CQ.

## Conclusions and prospects

ESA treatment for renal anemia in pre-dialysis CKD patients with a history of cancer can be considered, while taking into consideration patients’ wishes and when the benefits of treatments such as reduced amount of blood transfusion and iron replacement outweigh the harmful effects of thromboembolism and recurrence/new occurrence of cancer, following a careful course of treatment and monitoring to avoid high Hb levels. The descriptions in the existing guidelines are helpful in determining target Hb levels when diagnosing renal anemia and using ESA treatment [97, 103]. Additionally, when performing ESA therapy, it is necessary to pay attention to monitoring cancer recurrence and new occurrence.

No information on pediatric patients was available in this SR. Although HIF-PH inhibitors are effective as new therapeutic agents for renal anemia, they could not be examined here due to lack of information on their use by patients with a history of cancer. Through future research, we anticipate the establishment of evidence on the usage of therapeutic agents for renal anemia such as ESAs and HIF-PH inhibitors to maximize the beneficial effects and minimize the harmful effects in CKD patients with a history of cancer.

## GPS 1: Should carboplatin dosing be based on kidney function?

When administering carboplatin to adult patients with cancer, the target area under the curve (AUC) should be established and the dose should be determined based on kidney function. Although there are no papers evaluating the evidence that this kidney function-based dosing method enhances therapeutic efficacy and reduces side effects compared to the common method based on body surface area, this method is reasonable and widely used in clinical trials and routine clinical practice. (100% consensus, 27 voting, 27 agreeing).

## Background and purpose

The clearance of carboplatin is strongly correlated with GFR [104]. In addition, linearity has been observed between the AUC of free carboplatin and dosage [105]. The AUC, a measure of drug exposure in the body, correlates well with hematologic toxicity and antitumor effects. Therefore, the method of determining the dose based on the GFR after setting the target AUC is widely used. In the Calvert formula, the GFR value is often substituted for the CCr value. In this section, we examine the validity of determining carboplatin dosage based on the kidney function, as routinely used in clinical practice.

## Explanation

## Dose setting using the Calvert formula

Carboplatin is a platinum preparation used as a standard treatment for both lung and gynecological cancers. The clearance of carboplatin is strongly correlated with GFR [104], and a linearity between AUC and dose of free carboplatin has been observed [105]. Based on these pharmacokinetic characteristics, Calvert et al. developed a formula (Table 1) to calculate the appropriate carboplatin dose from the patient’s measured GFR and target AUC [106].

**Table 1:** Carboplatin dosage adjustment method (Calvert formula)

D = Target AUC × (GFR + 25)
D: Dose (mg), AUC: Area under the blood concentration curve (mg/mL × min)

Table created based on Calvert et al*.* J Clin Oncol 1989; 7: 1748–56 ^3^.^)^

A study analyzing AUCs calculated backward using the Calvert formula in patients with ovarian cancer treated with carboplatin at doses based on body surface area showed that the antitumor effect of carboplatin nearly plateaued at AUC values of 5–7 mg/min/mL, while hematologic toxicity, such as thrombocytopenia, showed a sigmoidal increase in AUC values of approximately 17 mg/min/mL [107]. For this reason, the Calvert formula is frequently used to calculate carboplatin dosages, which are often set at target AUC values of 5–7 mg/min/mL in the conventional treatment of many solid tumors. While both Egorin et al. [108, 109] and Chatelut et al*.* [110] have also developed carboplatin dosage formulas based on the kidney function, they are not as widely used because of the complexity of the calculations. In any case, although the method of setting a target AUC and determining the dose based on GFR is considered reasonable, there are no prospective clinical trials comparing it with the method of determining the dose based on body surface area. Indeed, there are not enough publications to evaluate the evidence.

## Substitution of GFR with CCr values

In the process of developing the Calvert formula, the actual GFR was measured by the clearance of EDTA labeled with the Cr radioisotope ^51^Cr. In Japan, inulin clearance, the standard method of measuring GFR, can be measured using National Health Insurance-covered procedures. However, this is often considered too complicated and burdensome for patients and healthcare professionals. As a result, carboplatin dosage is often calculated in clinical practice by substituting the Calvert formula GFR value for the CCr value.

In measuring CCr, the enzyme method has been used by most medical facilities in Japan since the mid-1990s. Thus, there is a risk of overdosage of carboplatin if the Calvert formula GFR value is substituted for the CCr value obtained in this way. Therefore, it has been proposed to use the CCr value obtained by adding 0.2 to the enzymatic serum Cr value and correcting it to the serum Cr value obtained by the Jaffe method [111]. Meanwhile, as the “GFR Estimation Formula for Japanese Patients” was published by the Japanese Society of Nephrology in 2008 [112], it is now possible to use an eGFR obtained from this estimation formula in the Calvert formula. However, as the eGFR value obtained by this estimation formula is a value per 1.73 m^2^ of standard body surface area (BSA, mL/min/1.73 m^2^), the GFR value (mL/min/body) for each patient must be obtained by multiplying eGFR value by BSA/1.73 when using the Calvert formula.

## Measurement of CCr

In the United States, serum Cr levels used to be measured by the Jaffe method. However, by the end of 2010, the method of measuring serum Cr levels was shifted to a method based on isotope dilution mass spectrometry (IDMS), allowing true serum Cr levels to be used in clinical practice. As a result, the serum Cr level decreased by approximately 0.2 mg/dL, and the dose of carboplatin was overestimated when the CCr value was substituted for the GFR value in the Calvert formula as in the past. Accordingly, the US Food and Drug Administration (FDA) established an upper limit of GFR (125 mL/min) to be used in the Calvert equation and stated that the upper limit of carboplatin dosage for AUC of 4, 5, and 6 mg/min/mL should be 600, 750, and 900 mg, respectively, to avoid carboplatin overdosage. A lower limit for serum Cr may also be set to prevent overdosage.

Some recent clinical trials did not use GFR values in the Calvert equation. For example, the KEYNOTE-189 trial (carboplatin, pemetrexed, and pembrolizumab) [113] and the KEYNOTE-407 trial (carboplatin, paclitaxel, and pembrolizumab) [114] on patients with non-small cell lung cancer. In these studies, the dose was calculated using the CCr value obtained by the enzyme method in the Calvert formula. To date, when substituting the enzymatic CCr value for the Calvert GFR value, the serum Cr value is corrected to the Jaffe Cr value by adding 0.2. However, in clinical trials such as the KEYNOTE-189 and KEYNOTE-407 trials, where CCr values were used in the Calvert formula, the carboplatin dose was determined according to the method used in those trials.

## Body size, kidney function, and clearance

The “GFR + 25” in the Calvert formula corresponds to the total clearance of carboplatin, of which “GFR” corresponds to renal clearance and the constant “25” to nonrenal clearance. However, nonrenal clearance depends primarily on body size. When the Calvert formula, developed in the United Kingdom, is used for Japanese patients, whose average body size is smaller than that of Caucasians, the nonrenal clearance is smaller than the constant “25,” reportedly to be “15” instead [115].

Although the Calvert formula is intended for patients with a GFR between 33 and 135 mL/min, it was originally derived from a study population in which the GFRs of the majority for patients were distributed in the range of 60–100 mL/min. Therefore, it is expected that the calculated doses will be less accurate for patients with GFRs outside this range. It is also unclear whether this formula can be used accurately in patients with severely impaired kidney function. Furthermore, nonrenal clearance has been reported to be smaller than the constant “25” [116]. Therefore, it should be noted that carboplatin may be overdosed in patients with severely impaired kidney function because the ratio of nonrenal clearance is relatively high compared to GFR.

## Issues when designing carboplatin dosing

In addition to the Cockcroft-Gault formula, there is the Jelliffe formula for estimating CCr; the Western MDRD, CKD-EPI, and Wright formulas for estimating GFR; as well as the Japanese GFR estimation formula (eGFR). When using these estimation formulas, it is necessary to consider differences in patient backgrounds, such as ethnicities and clinical conditions, as well as differences in serum Cr measurement methods. Since use of these estimation formulas assumes that serum Cr levels are stable, kidney function is overestimated when kidney function fluctuates greatly, such as in the acute phase of renal failure, or when muscle mass is severely reduced, such as in sarcopenic or malnutrition.

When interpreting carboplatin clinical trials, it is important to take into account the method used to evaluate serum Cr values, the formula used to determine the value substituted for GFR in the Calvert calculation, and any specified upper limits of the GFR or lower limits for the serum Cr values. As the estimated values may differ greatly depending on each calculation method, it is unclear to what extent the type of calculation formula used affects the actual clinical efficacy.

## GPS 2: Should dialysis be performed to remove drug after administering cisplatin to patients on maintenance hemodialysis?

Most tissue- and protein-bound cisplatin remains in the body after hemodialysis, and after dialysis, a rebound blood level is observed to rise again. Therefore, hemodialysis therapy for drug removal should not be performed after cisplatin administration for patients on maintenance hemodialysis. (100% agreement, 27 voting, 27 agreeing).

## Background and purpose

In patients undergoing maintenance hemodialysis, the drug may accumulate in the body after cisplatin administration and produce adverse effects. Dialysis may be used to mitigate these adverse effects. In this section, we evaluated the efficacy of dialysis therapy for the purpose of drug elimination, after cisplatin administration.

## Explanation

## Cisplatin pharmacokinetics

Cisplatin is an injectable anticancer drug that exerts its antitumor effect by inhibiting DNA replication and transcription by forming cross-links within and between DNA strands. Approximately 4 h after administration, more than 90% of cisplatin is irreversibly covalently bound to plasma proteins. The antitumor activity and toxicity of cisplatin are due to the plasma protein-unbound or free form of cisplatin. While cisplatin is transported in high concentrations to the kidney, liver, intestinal tract, and testes, it is poorly transferred to the brain. Although cisplatin is renally excreted with little biliary excretion or secretion from the intestinal tract, the cumulative urinary recovery rate of cisplatin after a single administration is approximately 30%, even at 5 days after the end of administration, indicating that the majority of the drug remains in the body. For patients receiving hemodialysis whose kidney function has already been destroyed, cisplatin-induced kidney impairment is not a problem. However, incidences of bone marrow toxicity, gastrointestinal toxicity, and neurotoxicity require attention.

## Removal of free form cisplatin by hemodialysis after administration

Drugs that do not bind to plasma proteins and are primarily distributed within blood vessels (low protein binding rate) are easily removed by dialysis. Moreover, the smaller the molecular weight of the drug, the more likely it is to be removed by hemodialysis. There have been case reports of promising results with cisplatin administered to patients on hemodialysis 30‒60 min after administration [117–119]. If the free form of cisplatin with the antitumor effect is removed by hemodialysis after administration, the effect may be weakened. However, as cisplatin acts nonspecifically in the cell cycle, its effect may occur even without continuous administration of high concentrations of cisplatin. Therefore, even though performing hemodialysis 30‒60 min after cisplatin administration is presumed reasonable from the viewpoint of efficacy and safety, there are no publications to evaluate the evidence for this. The recommendations of the Italian Society of Oncology and the Italian Society of Nephrology state that “dialysis should be performed 60 min after cisplatin administration” when a cisplatin-containing combination therapy is administered to patients undergoing hemodialysis [120]. In contrast, these guidelines also state that “the dosage should be reduced to 25–50% after hemodialysis” because even if free form cisplatin is rapidly removed by hemodialysis, the dissociation of protein-bound cisplatin will not compensate for this reduction [120]. In any case, the recommendations of the Italian Society of Oncology and the Italian Society of Nephrology do not clearly state the benefits and risks of hemodialysis for the purposes of drug removal after cisplatin administration.

Few studies, other than case reports, have systematically investigated the pharmacokinetics of cisplatin administered to patients on hemodialysis. Miyagawa et al. reported the pharmacokinetic results of cisplatin administered to five patients who developed gastric cancer during maintenance dialysis. When cisplatin was administered simultaneously from the start of dialysis, the blood concentration of free cisplatin decreased rapidly, and after passing through the dialyzer, the blood concentration was below the sensitivity level. In contrast, the blood concentration of bound cisplatin showed a relatively steep initial change, followed by a gradual decrease. The blood concentrations of free and bound cisplatin were virtually identical even when hemodialysis was started 1 h after administration. However, in which of the five cases the cisplatin was administered at the same time as the start of hemodialysis and in which cases, dialysis was started 1 h after cisplatin administration was not specified [121]. In the same year, Miyagawa et al. also reported the pharmacokinetics of cisplatin in two patients with gastric cancer on maintenance hemodialysis, yet it is unclear whether they were included in the five cases reported above [122].

## Concentration re-elevation due to rebound cisplatin

While free cisplatin can be removed by hemodialysis, bound cisplatin is difficult to remove and leads to toxicity. As such, it has been reported that some physicians have reduced the dose to 50–75% in patients on dialysis [123]. Even when hemodialysis is performed for approximately 3.5–4 h after cisplatin administration to remove the drug, the free cisplatin is primarily removed, which is approximately 10% of the total cisplatin administered [124]. Therefore, even when hemodialysis is performed after administration, attention should be paid to the accumulation of cisplatin. After hemodialysis, the blood concentration of free cisplatin increases again due to rebound [117]. In addition, the removal rate of cisplatin by hemodialysis has been reported to decrease as the cumulative amount of cisplatin increases [123, 125].

Thus, even though free cisplatin is removed by hemodialysis, most of the tissue- and protein-bound cisplatin remains in the body and blood levels are expected to rise again after dialysis due to rebound. Thus, in agreement with the previous guideline, the authors propose in this GPS 2 that “hemodialysis therapy for drug removal should not be performed after cisplatin administration in patients on maintenance hemodialysis.”

## Future investigation

As it is difficult to measure the blood concentration of cisplatin, platinum concentration is often measured by atomic absorption spectroscopy as an alternative method. Specifically, measurement of protein-bound platinum is substituted for bound cisplatin and that of unbound platinum is substituted for free cisplatin. However, as it is unknown whether the structure of cisplatin is maintained or degraded in both the bound and free forms, although the concentration trend of platinum is correct, whether the concentrations of bound and free cisplatin indicate correct values remains unclear. Further information should be obtained by measuring the blood concentration of cisplatin and the activity of cisplatin degradation products in the future.

Although there are reports of cisplatin administration in patients on hemodialysis with dose reductions of 50–75% [123], there are also reports of cisplatin administration at full dose [126]. The benefit of cisplatin administration at full dose to avoid loss of dose-intensity and performing hemodialysis immediately after administration to minimize adverse effects are also topics for further investigation.

## GPS 3 Is growth hormone therapy recommended for childhood cancer survivors (CCS) with CKD?

The use of growth hormone should be considered for height gain in CCS with CKD. However, there is insufficient evidence regarding the risk of developing secondary cancer with growth hormone therapy, and the decision to use it should be made after careful consideration of the balance of benefits and harms. (100% agreement, 27 votes, 27 in favor).

## Background and Purpose

Impaired physical development is a clinically important problem in both pediatric CKD patients as well as CCS. The use of recombinant human growth hormone (rhGH) has been considered to achieve height gain, and its use is recommended, especially in the area of pediatric CKD [103]. In Japan, rhGH is actively used in pediatric CKD patients with impaired physical development when indicated. However, due to its cell proliferative effects, especially in CCS, attention should be paid to the possibility of tumor recurrence and the development of secondary cancers.

## Explanation

## Efficacy

Risk factors for growth hormone deficiency in CCS include brain tumors, therapeutic cranial irradiation, and surgical operations involving the hypothalamus and pituitary gland [127]. There are several reports which have examined the efficacy of rhGH for growth hormone insufficiency due to tumor or treatment [128–130].

In a meta-analysis of 29 studies reported in 2018, rhGH use was associated with height gain in CCS. The authors reported a height gain of + 0.95 standardized mean difference (SD) (95% CI 0.18–1.72) in patients who received rhGH compared to those who did not [129]. In addition, an observational study of 87 CCS treated with rhGH reported in 2020 concluded that although only 1/3 of the patients were able to reach the target height, the mean final height was − 0.85 SD, meaning that the treatment was effective in preventing more severe impairment of height gain [128].

## Safety

While its effectiveness to height gain, awareness is required for the possibility of tumor recurrence and the development of secondary cancers such as meningioma owing to the cell proliferative effects of growth hormone, especially in CCS.

The meta-analysis described above showed no clear increased risk of cancer recurrence, with an odds ratio (OR) of 0.57 (95% CI 0.31–1.02) for recurrence with growth hormone use compared to no use, but the OR for secondary cancer development was 1.34 (95% CI 0.92–1.96) (The OR presented in the text of the paper differs from that in the figure, and we confirmed that the OR shown here is correct by contacting the corresponding author.) [129].

In a 26-year observational study of CCS reported in 2020, growth hormone use was not clearly associated with the development of a secondary cancer, with a 1.3-fold risk (95% CI 0.9–2.0) compared to non-use [131]. However, an effect size of approximately 1.3 for the critical outcome of cancer development may not be concluded as completely safe even though statistical significance was not found.

## Initiation criteria and dosage

It is difficult to draw conclusions regarding safe rhGH initiation criteria and dosage in CCS with CKD.

In Japan, the initiation criteria of rhGH in CKD are that the epiphyseal line is not closed, and that the height of the patient is − 2 SD or less of the same age. The dosage covered by Japanese National Health Insurance is set at 0.175–0.35 mg/kg/week, which is higher than that for growth hormone deficiency, because CKD is a GH-refractory condition. In addition, to prevent cancer recurrence, the conventional practice with CCS patients is to start rhGH administration 1 year after remission in Europe and the United States, and at least 2 years after remission in Japan [127, 132].

However, the relationship between rhGH administration and cancer recurrence is not clear, and there is a lack of evidence regarding the safe period between remission and the start of rhGH administration [132, 133]. In the Safety and Appropriateness of Growth Hormone Treatments in Europe (SAGhE) cohort study of > 10,000 rhGH-treated patients including approximately 1,800 CCS, conducted over 26 years in eight European countries, 37 of the 38 patients who developed meningiomas during the observation period were CCS. This report examined risk factors for meningioma development among CCS who received radiotherapy, concluding that age at initiation of rhGH, administration period, and dose (daily dose, cumulative dose) did not correlate with meningioma development [134]. There have been no reports to date examining cancer recurrence or secondary cancer development due to an overdose of rhGH in CCS.

## Summary

Height gain is one of the important outcomes in CCS with CKD, and the use of rhGH may improve height gain. However, rhGH should only be administered after careful consideration of the possibility of secondary cancer development, especially after kidney transplantation, because of long-term immunosuppression. Therefore, when rhGH is used, periodic screening for tumors might be considered. Patients’ wish for height gain is an important factor in the decision to use rhGH.

## GPS 4 What is the appropriate kidney replacement therapy for childhood cancer survivors?

The most preferred kidney replacement therapy in children with CKD is kidney transplantation. Although there is insufficient evidence regarding the choice of kidney replacement therapy in CCS, kidney transplantation should be the first choice after an appropriate waiting period following cancer treatment (100% agreement rate, 27 votes, 27 in favor).

## Background and purpose

The risk of end stage kidney disease requiring kidney replacement therapy during young adulthood in CCS is reported to be approximately 9 times higher than that of non-CCS [135]. Therefore, selecting the optimal kidney replacement therapy for CCS after the completion of drug therapy is a critical issue. Kidney transplantation is recommended as the first choice of kidney replacement therapy in children with CKD [136]. However, because long-term immunosuppression after kidney transplantation is a potential risk factor for the development of cancer, careful consideration is required for CCS. In addition, the timing of kidney transplantation also requires careful consideration from the perspective of recurrence, secondary cancers, and survival, although it is typically performed 2–3 years after cancer treatment in Japanese clinical practice.

## Explanation

## Prognosis

No literature comparing kidney transplantation and dialysis in CCS in terms of clinical outcomes is available.

According to a relatively large study of patients undergoing solid organ transplantation from the United States, the 5-year survival rate after kidney transplantation was 93.5% among CCS, which was not different from the overall 5-year survival rate of 95.5% for kidney transplant recipients [137]. In a study of patients with Wilms’ tumor (nephroblastoma), transplant patients had a much lower mortality rate than dialysis patients (hazard ratio = 0.16, 95% CI 0.07–0.38) though the result was not obtained by the main analysis, the confounders were not adjusted, and some patients died before kidney transplantation which may result in potential survivor bias [138]. This study also examined the risk of developing secondary cancer after kidney transplantation; however, the number of events was too small to provide sufficient results. Recurrence after kidney transplantation was observed in 1 out of 117 patients.

## Indication

The indication for kidney transplantation for CCS requires more careful consideration than for non-CCS because long-term immunosuppression may be a risk factor for cancer development. However, it may be beneficial, especially for survival, growth, development, and QOL [139]. Thus, kidney transplantation should be selected as kidney replacement therapy.

## Timing of transplantation

When is the best time for transplantation? Many studies on this topic have been limited to patients with Wilms’ tumor, who develop kidney failure early after cancer treatment. Since two case series were published in 1979, the waiting period for kidney transplantation has conventionally been 1 to 2 years after cancer treatment. One of these studies included 20 patients who underwent kidney transplantation after treatment for Wilms’ tumor. In this study, 7 of 15 patients who underwent kidney transplantation < 1 year after cancer treatment had recurrence or metastasis, whereas no recurrence or metastasis was observed in patients who underwent kidney transplantation 1 year after treatment or later [140]. The other study was a case series of 26 patients with Wilms’ tumor consisting of 17 patients who underwent kidney transplantation and 9 patients who did not. Although death due to sepsis was notably more frequent in transplant patients (11% in non-transplant patients and 53% in transplant patients), 3 of the 5 survivors received transplantation 1 year after cancer treatment [141].

To date, there have been no interventional trials examining waiting period and outcomes. Previous observational studies showed a mortality risk of 0.9 (95% CI 0.3–3.3) and 0.6 (95% CI 0.1–2.6) for patients undergoing kidney transplantation 0–1 year and 1–2 years after Wilms’ tumor treatment, respectively, compared to ≥ 2 years [138]. However, because of the small number of events, these results do not imply that transplantation within 2 years is risk-free.

In a cohort study of patients who underwent kidney transplantation after treatment for Wilms’ tumor, the risk of death was not different from that of patients without Wilms’ tumors. Because more than half of the patients in this cohort underwent kidney transplantation > 1 year after cancer treatment, the authors concluded that the results support the previously recommended waiting period of 1 to 2 years [142].

Despite these few observational studies, there is little evidence to support a waiting period of 1 to 2 years between cancer treatment and kidney transplantation for patients with kidney failure after treatment for Wilms’ tumor. An accumulation of evidence is expected in the future.
