# Correction to: Clinical questions and good practice statements of clinical practice guidelines for management of kidney injury during anticancer drug therapy 2022

**DOI:** 10.1007/s10157-023-02436-9

**Published:** 2023-12-09

**Authors:** Motoko Yanagita, Satoru Muto, Hiroyuki Nishiyama, Yuichi Ando, Sumio Hirata, Kent Doi, Yutaka Fujiwara, Norio Hanafusa, Takahiro Hatta, Junichi Hoshino, Satoko Ichioka, Takamitsu Inoue, Kenji Ishikura, Taigo Kato, Hiroshi Kitamura, Yusuke Kobayashi, Yuichi Koizumi, Chihiro Kondoh, Takeshi Matsubara, Kazuo Matsubara, Koji Matsumoto, Yusuke Okuda, Yuta Okumura, Emiko Sakaida, Yugo Shibagaki, Hideki Shimodaira, Nao Takano, Akiko Uchida, Kimikazu Yakushijin, Takehito Yamamoto, Kazuhiro Yamamoto, Yoshinari Yasuda, Mototsugu Oya, Hirokazu Okada, Masaomi Nangaku, Naoki Kashihara

**Affiliations:** 1 Department of Nephrology, Kyoto University Graduate School of Medicine, Kyoto, Japan; 2 Institute for the Advanced Study of Human Biology (ASHBi), Kyoto University, Kyoto, Japan; 3 Department of Urology, Graduate School of Medicine, Juntendo University, Bunkyo City, Tokyo Japan; 4 Department of Urology, Faculty of Medicine, University of Tsukuba, Tsukuba, Ibaraki Japan; 5 Department of Clinical Oncology and Chemotherapy, Nagoya University Hospital, Nagoya, Japan; 6 Department of Academic Education, I and H Co., Ltd, Ashiya, Japan; 7 Department of Emergency and Critical Care Medicine, The University of Tokyo Hospital, Bunkyo City, Tokyo Japan; 8 Department of Thoracic Oncology, Aichi Cancer Center, Nagoya, Japan; 9 Department of Blood Purification, Tokyo Women’s Medical University, Shinjuku City, Tokyo Japan; 10 Department of Respiratory Medicine, Anjo Kosei Hospital, Anjo, Japan; 11 Department of Nephrology, Tokyo Women’s Medical University, Shinjuku City, Tokyo Japan; 12 Department of Pediatrics, Shiga University of Medical Science, Otsu, Shiga Japan; 13 Department of Renal and Urologic Surgery, International University of Health and Welfare Narita Hospital, Chiba, Japan; 14 Department of Pediatrics, Kitasato University School of Medicine, Minato, Kanagawa Japan; 15 Department of Urology, Osaka University Graduate School of Medicine, Osaka, Japan; 16 Department of Urology, Faculty of Medicine, University of Toyama, Toyama, Japan; 17 Department of Obstetrics and Gynecology, Keio University School of Medicine, Minato, Tokyo Japan; 18 Department of Pharmacy, Seichokai Fuchu Hospital, Izumi, Japan; 19 Departments of Medical Oncology, National Cancer Center Hospital East, Kashiwa, Japan; 20 Department of Pharmacy, Wakayama Medical University Hospital, Wakayama, Japan; 21 Hyogo Cancer Center, Akashi, Hyogo Japan; 22 Department of Gastrointestinal and Medical Oncology, National Hospital Organization Kyushu Cancer Center, Fukuoka, Japan; 23 Department of Hematology, Chiba University Hospital, Chiba, Japan; 24 Division of Nephrology and Hypertension, Department of Internal Medicine, Saint Marianna University School of Medicine, Kawasaki, Kanagawa Japan; 25 Division of Medical Oncology, Faculty of Medicine, Tohoku Medical and Pharmaceutical University, Sendai, Japan; 26 Department of Gastroenterological Surgery (Surgery II), Nagoya University Graduate School of Medicine, Nagoya, Japan; 27 Department of Nursing, Seirei Sakura Citizen Hospital, Chiba, Japan; 28 Department of Medical Oncology and Hematology, Kobe University Hospital, Kobe, Hyogo Japan; 29 Department of Pharmacy, The University of Tokyo Hospital, Bunkyo City, Tokyo Japan; 30 Department of Pharmacy, Kobe University Hospital, Kobe, Japan; 31 Department of Nephrology, Internal Medicine, Nagoya University Graduate School of Medicine, Nagoya, Japan; 32 Department of Urology, Keio University School of Medicine, Minato, Tokyo Japan; 33 Department of Nephrology, Saitama Medical University, Saitama, Japan; 34 Division of Nephrology and Endocrinology, The University of Tokyo, Bunkyo City, Tokyo Japan; 35 Department of Nephrology and Hypertension, Kawasaki Medical School, Kurashiki, Japan

**Correction to: Clinical and Experimental Nephrology** 10.1007/s10157-023-02415-0

In the original publication, the abstract text has been inadvertently included in the article. We have removed the abstract text in the updated version.

The original article has been corrected.

